# Synthesis, molecular docking study, MD simulation, ADMET, and drug likeness of new thiazolo[3,2-*a*]pyridine-6,8-dicarbonitrile derivatives as potential anti-diabetic agents

**DOI:** 10.1371/journal.pone.0306973

**Published:** 2024-09-12

**Authors:** Fatemeh Aghahosseini, Mohammad Bayat, Zahra Sadeghian, Davood Gheidari, Fatemeh Safari

**Affiliations:** 1 Department of Chemistry, Faculty of Science, Imam Khomeini International University, Qazvin, Iran; 2 Department of Chemistry, Faculty of Science, University of Guilan, Rasht, Iran; Babasaheb Bhimrao Ambedkar University (A Central University), INDIA

## Abstract

There are numerous uses for the pharmacological effects of thiazolo-pyridine and its derivatives. The main objective of the study was to synthesis 10 novel derivatives of thiazolo[3,2-*a*] pyridine-6,8-dicarbonitrile with a 22–78% yield, with a focus on their potential anti-diabetic properties. We investigated the interactions between these compounds and the enzyme α-amylase through an in *silico* study involving molecular docking. According to the docking analysis results, the resulting compounds had advantageous inhibitory properties. With a docking score of -7.43 kcal/mol against the target protein, compound **4e** performed best. The stability root-mean-square deviation (RMSD) showed that the complex stabilizes after 25 ns and with minor perturbation at 80. The RMSF values of the ligand-protein complex indicate that the following residues have interacted with compound 4e during the MD simulation: Trp58, Trp59, Tyr62, Gln63, His101, Val107, lle148, Asn152, Leu162, Thr163, Gly164, Leu165, Asp197, Ala198, Asp 236, Leu237, His299, Asp300, and His305. Moreover, the pharmacokinetic and drug-like properties of the synthesized derivatives of 2-arylamino-dihydroindeno[1,2-*b*] pyrrol-4(1*H*)-one suggest that they have the potential to be effective inhibitors of α-amylase and should be considered for further research. Nevertheless, it is crucial to ascertain the in *vivo* and in *vitro* effectiveness of these compounds through biochemical and structural investigations.

## 1. Introduction

**Fig 1:**
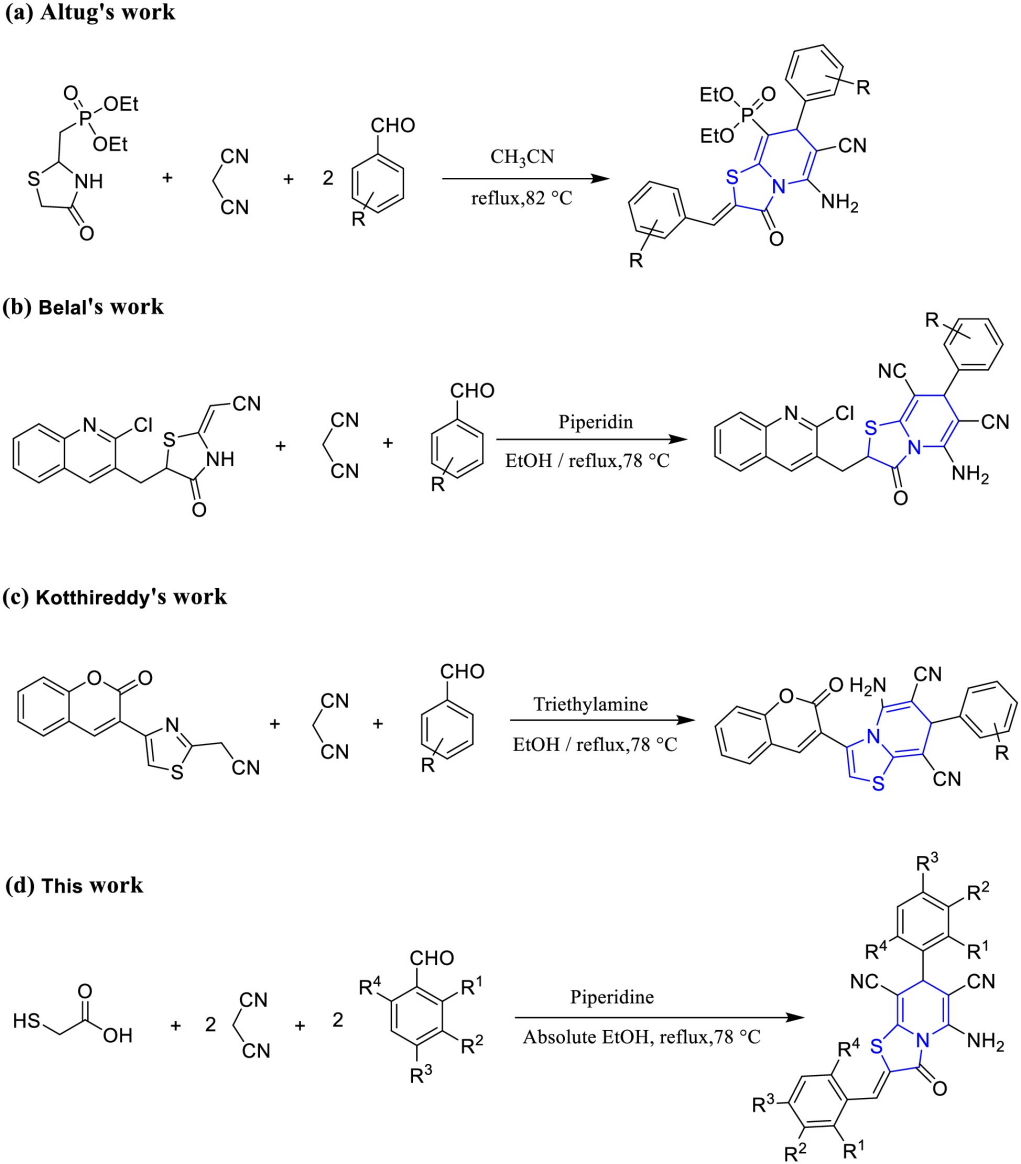
Previous and present works of thiazolo[3,2-*a*]pyridine synthesis.

Diabetes mellitus is a long-term metabolic condition marked by increased levels of glucose in the bloodstream due to insufficient insulin production or utilization within the body [1, 2]. Sustained high blood sugar levels can give rise to numerous complications that significantly impact both physical well-being and overall quality of life [3]. T2DM is the predominant form of diabetes, making up around 85% to 90% of all diagnosed cases [4, 5]. Pancreatic α-amylase serves as a significant enzyme involved in starch breakdown within the small intestine. Its primary function is to break down dextrinized starch into maltose and various oligosaccharides by catalyzing the hydrolysis of the α-1,4-glucosidic bond. The subsequent digestion of the produced substances occurs through the action of other glucosidases [6]. A successful strategy to mitigate the effects of T2DM involves the inhibition of α-amylase in the gastrointestinal tract, leading to a delay in the digestion of starchy foods. This, in turn, effectively suppresses postprandial hyperglycemia and reduces its impact on individuals with T2DM. Studies have indicated that thiazolopyridines, which consist of two fused heterocyclic rings, exhibit inhibitory effects on enzymes associated with diabetes [7]. Due to the diverse biological properties of thiazolopyridine structures, they have garnered significant interest as promising synthetic targets, with numerous reports available in the literature. For instance, thiazolo[3,2-*a*]pyridin-8-yl-phosphonate, which serve as a representative of plant growth regulators, were synthesized by the reaction of diethyl (*E*)-(4-oxothiazolidin-2-ylidene)methyl)phosphonate, malononitrile, and aromatic aldehyde derivatives (Fig 1a). This work was reported by Altug *et al*. in 2019 [8]. Belal *et al*. developed the synthesis of thiazolo[3,2-*a*] pyridine-6,8-dicarbonitrile structures through a reaction of 2-chloro-quinoline-3-carbaldehyde, 2-(4-oxo-4,5-dihydrothiazol-2-yl)acetonitrile, malonitrile, and aromatic aldehydes in EtOH, in the presence of a catalytic amount of piperidine (Fig 1b) [9]. Kotthireddy *et al*. studied the formation of these structures using 2-(4-(2-oxo-2H-chromen-3-yl) thiazol-2-yl) acetonitrile as a crucial precursor. They revealed that target compounds can be obtained in a stepwise manner and a tandem method too (Fig 1c) [10]. Fettach *et al*. have shown that benzylidene-thiazolidinediones have inhibitory activity against α-amylase, with an IC50 value of 18.19 ± 0.11 μM [11]. In their 2022 study, Khan *et al*. discovered that thiazolidinone-based indole compounds had inhibitory effects on α-amylase, with an IC50 value of 1.80 ± 0.70 μM [12]. In another investigation, they showed that enzimidazoles containing thiazolidinone derivatives (IC50 value: 2.10 ± 0.10 μM) are α-amylase inhibitors [13]. Over the past decade, there has been a consistent increase in the rate of drugs failing in the later stages of clinical trials. This is because the assumptions developed for rational drug design, such as the idea of ’one drug, one gene, one disease’, were based on the belief that this approach would result in safer and more effective drugs. The goal was to design target-selective ligands that would eliminate undesirable side effects. Nevertheless, this idea has been consistently challenged throughout the last decade. The advancements in bioinformatics, polypharmacology, and network pharmacology have garnered interest as a cost-effective strategy for drug discovery, focusing on several targets simultaneously [14]. Alegaon *et al*. recently reported the synthesis of benzene sulphonamide-thiazolidin-4-one hybrids as promising efficacy against α-amylase inhibition with an IC50 value of 29.51±1.35 μg/ml [15]. Network pharmacology may improve knowledge of lead molecules and their interactions with targets, resulting in greater insights into the development of antidiabetic drugs. In order to induce a pharmacological response, structural modifications involving molecular framework modifications of compounds are required, according to the aforementioned research and literature. Herein, we carried out a one-pot, three-component process to synthesize a series of thiazolo[3,2-*a*]pyridine-6,8-dicarbonitrile derivatives (Fig 1d). By employing the molecular docking method, conducting ADMET analysis, and evaluating their drug-like characteristics, we have provided evidence of the inhibitory potential of these compounds against α-amylase.

## 2. Result and discussion

### Synthesis

**Fig 2:**
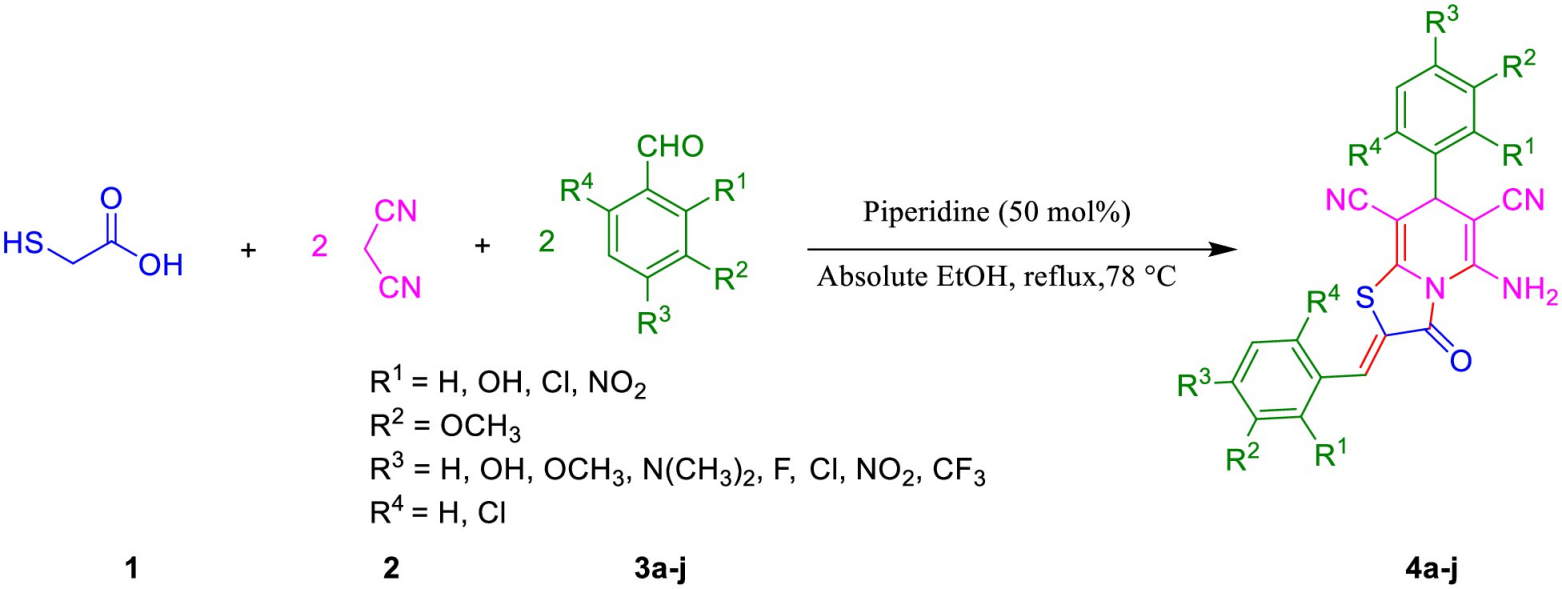
Synthesis of thiazolo[3,2-*a*] pyridine-6,8-dicarbonitrile derivatives.

We synthesized a series of new functionalized thiazolo[3,2-*a*]pyridine-6,8-dicarbonitrile structures **4a–j** by using thioglycolic acid **1,** two equivalents of malononitrile **2,** and various aromatic aldehydes **3a–j** in absolute EtOH at reflux conditions (Fig 2).

The optimized reaction conditions were determined by using thioglycolic acid **1**, two equivalents of malononitrile **2**, and 4-chlorobenzaldehyde **3a** as model substrates, in the presence of a piperidine catalyst. With the point of green chemistry, water solvent was tested in this reaction, but any product was not observed, neither at room temperature nor at the reflux condition (Table 1, entries 1–2). Then, EtOH was used, and the product was obtained after adding crushed ice and diluted HCl with a 52% yield (Table 1, entry 3). The product yield in the mixture of water / EtOH was very low (Table 1, entry 4). It was found that water or a ratio of water in the solvent mixture cannot proceed this reaction well. The reaction was conducted in absolute EtOH and resulted in a high yield without the need for any further purification processes (Table 1, entry 5). To obtain the best amount of piperidine as a catalyst, several percentages were tested (Table 1, entries 6–10). The efficiency of piperidine was confirmed by comparison with triethylamine as a catalyst in this work (Table 1, entry 11). According to these results, absolute EtOH at reflux and 50% piperidine were applied as optimized reaction conditions. Having obtained the most favorable reaction conditions, the procedure was expanded, resulting in the preparation of a range of thiazolo[3,2-*a*] pyridine-6,8-dicarbonitrile derivatives. Table 2 summarizes these results. Based on these results, it seems that the steric and electronic features of substitutes on the aromatic aldehyde ring play a role in this process and affect the product yield. For example, the highest product yield **4h** was obtained when 4-nitrobenzaldehyde was used (78%). Clearly, the -NO_2_ group as an electron-withdrawing group makes the electrophile aldehyde moiety undergo a nucleophile such as malononitrile at the first step of this process. Using 4-dimethylaminobenzaldehyde led to the lowest product yield **4j**. Obviously, -N(CH_3_)_2_ acts as an electron-donating group, which decreases the electrophilicity of the aldehyde moiety. Steric effects decrease the product yield in **4f** and **4c** in comparison with **4a**. The presence of a -Cl substitute in the *ortho* position hinders a nucleophile attack, and there is more hinderance when two *ortho* positions are occupied with substitutes.

**Table 1:** Optimizing reaction condition for 4a.

Entry	Solvent	Catalyst (50 mol%)	Temperature (° C)	Time (h)	Yield (%)
1	H_2_O	Piperidine	25	24	No Reaction
2	H_2_O	Piperidine	100	24	No Reaction
3	EtOH / HCl	Piperidine	78	24	52
4	EtOH / H_2_O	Piperidine	80	16	27
**5**	**Absolute EtOH**	**Piperidine**	**78**	**16**	**56**
6	Absolute EtOH	Piperidine (100%)	78	16	27
7	Absolute EtOH	Piperidine (30%)	78	16	50
8	Absolute EtOH	Piperidine (20%)	78	16	49
9	Absolute EtOH	Piperidine (10%)	78	16	39
10	Absolute EtOH	-	78	16	32
11	Absolute EtOH	Et_3_N	78	18	47

**Table 2:** Synthesizing 4a-j derivatives.

Entry	Aldehyde	Products	Time (h)	Yield (%)
1	4-Cl-PhCHO	4a	16	56
2	2-NO_2_-PhCHO	4b	21	46
3	2,6-diCl-PhCHO	4c	17	30
4	4-F-PhCHO	4d	19	35
5	4-CF_3_-PhCHO	4e	24	58
6	2-Cl-PhCHO	4f	19	44
7	4-OH-PhCHO	4g	24	48
8	4-NO_2_-PhCHO	4h	18	78
9	2-OH-3-OMe-PhCHO	4i	23	30
10	4-NMe_2_-PhCHO	4j	22	22

**Fig 3:**
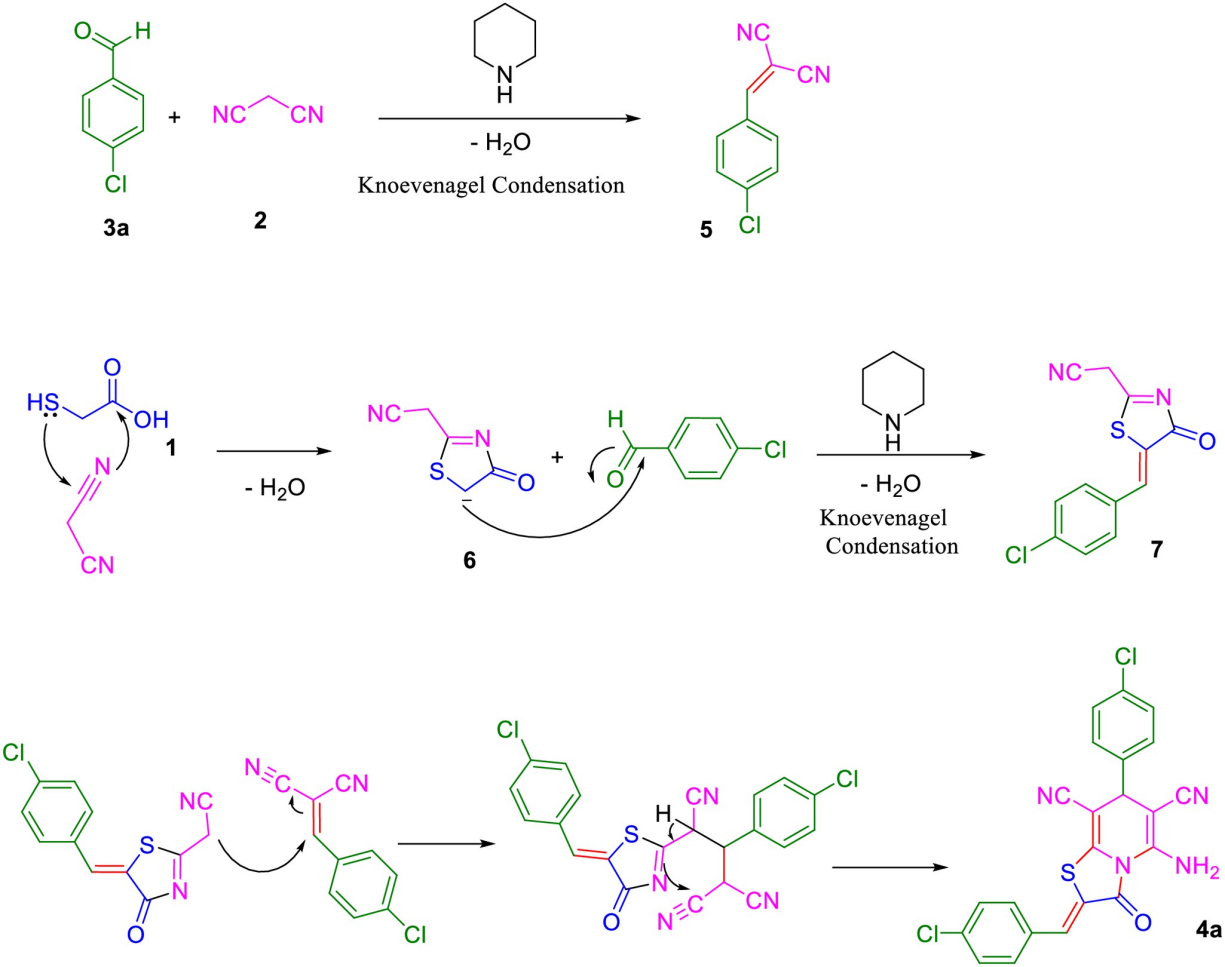
Possible mechanism for synthesizing 4a.

The possible mechanism reaction for synthesizing **4a** is shown in Fig 3. First, the Knoevenagel condensation reaction of one equivalent 4-chlorobenzaldehyde **3a** and malononitrile **2** leads to the formation of compound **5**. Simultaneously, product **6** was formed through a cyclization reaction of thioglycolic acid and another equivalent of malononitrile **2.** Then, this product undergoes another 4-chlorobenzaldehyde **3a** reaction to obtain compound **7**. Finally, the desired product **4a** is formed by the nucleophilic addition of **7** to **5**, followed by intramolecular cyclization.

### Molecular docking studies

Molecular docking is the process of identifying appropriate ligands that can fit both geometrically and energetically into a protein’s active site. We utilized PDB code 4W93 [16] to perform molecular docking investigations of synthesized molecules with α-amylase, and the active site of α-amylase is shown in Supporting Information. The docking process was confirmed by re-docking the co-crystal ligand into the active pocket of the α-amylase. The RMSD value was below 2 Å, indicating the reliability of the docking procedure [17]. The active site of the protein was effectively targeted by docking potent thiazolo[3,2-*a*] pyridine-6,8-dicarbonitrile derivatives to essential amino acid residues. To assess the effectiveness of the synthesized derivatives, their binding affinities and binding scores were evaluated, employing the specific amino acid residues found in the active pocket. The affinity score was utilized to assess the strength of binding between the compounds. The findings revealed that all of the compounds exhibited robust binding scores and outstanding binding affinities. Notably, among these compounds, compound **4e** displayed the highest binding energy, measured at -7.43 Kcal/mol. The best conformation of compound **4e** was selected, and both bonding and non-bonding interactions were thoroughly analyzed. The docking outcomes of the potent compounds, along with their respective interactions, are presented in Table 3.

**Table 3:** Binding affinity, and interaction modes compounds (4a–j) against α-amylase.

Compound	Docking Score(kcal/mol)	Interactions								
		H- bonding	C-bonding	π-Donor H-bonding	Van der walls	Hydrophobic	Attractive Charge	Halogen	Unfavorable	Other
**4a**	-7	Asp197	Asp300		Thr163,Gln63,Leu165,Tyr62,His101,His299,Ala198,Glu233	Ile235,Lys200, Trp59,Leu162, His201				
**4b**	-6.21	Trp59,His305	Asp356		Trp357,Trp58,Thr163,Gln63,Leu165,His201,Ile23,His299,Pro54		Asp356,His305,Asp300,Glu233,Asp197,Arg195			
**4c**	-6.85	Trp59	Asp300	Tyr62	Gln63,Leu165,His101,Asp197,Leu162,Arg195,Ile235,Trp58,Thr163,His305,Gly104	Trp59				
**4d**	-7.14	Gln63	Asp300, Gly104	His305	Glu233,Ile235,Trp58,Ala198,Leu162,Asp197,Arg195,Tyr62,His101,Leu165,Thr163,Gly164,Val107,Ala106,Ile51			Gly104	Trp59	His299
**4e**	-7.43	Lys200	Asp300, His201		Trp357,Thr163,His305,Ala198,Glu233,Asp197,His101,Trp58,His299,Tyr62,Leu165,Gln63	Trp59,His201,Ile235,Lys200,Tyr151,Leu162		Ile235		
**4f**	-6.29	Gln63	Asp300		Ile235,Leu162,Trp58,His305,Thr163,Gly104,Leu165,His101,Tyr62,Arg195,Asp197,Glu233,Gly306	Trp59			His299	
**4g**	-6.34	Gln63	Thr163,Asp300	His305,Tyr62	Ile235, Glu233, Trp58,Gly306,Gly104,Ala106,Val107,Gly164,Asn105,Leu165,His101,Asp197,Arg195, Leu162				His299	Trp59
**4h**	-6		Asp300		Tyr151,Ile235,His201,Trp58,Leu162,Glu233,Arg195,Asp197,Ala198,Tyr62,His10,Thr163,Gly104,Gln63,Ile51,His305,Gly306,	Trp59,Leu165			His299	
**4i**	-5.63	Glu233,Asp197	Asp300,Tyr151		Lys200,Arg195,His299,Asn298,Trp58,Gln63,Thr163,Leu165,Tyr62,His101,Ala198,Leu162,Ile235,His201					Trp59
**4j**	-5.90		Asp300,His201		Tyr151,Lys200,His299, Arg195, Glu233,Trp58,Asp197,Ala198,Tyr62,His101,Leu165,Thr163,Gln63, His305, Ile235	Trp59,Leu162				

**Fig 4:**
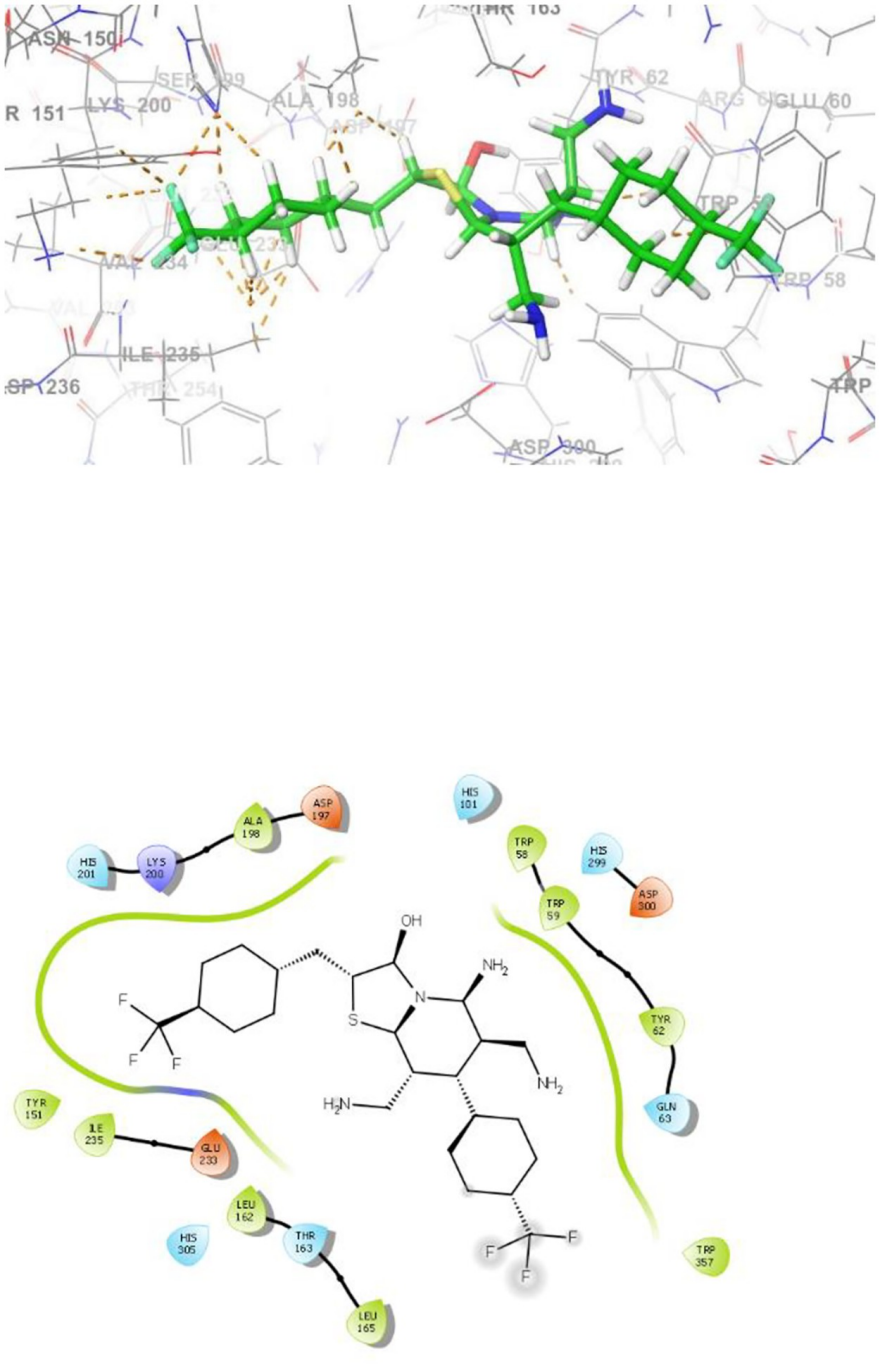
The 3D and 2D binding modes of 4e in the active pocket of α-amylase.

Fig 4 illustrates the detailed 3D and 2D interactions that compound **4e** engages in within the active site of α-amylase. The amino acid residues Lys200, Asp300, His201, Ile235, Trp59, Leu162, Tyr151, Trp357, Thr163, His305, Ala198, Glu233, Asp197, His101, Trp58, His299, Tyr62, Leu165, and Gln63 play a role in forming both bonding and non-bonding interactions with compound **4e**. In summary, compound **4e** exhibits a total of three hydrogen bond interaction. Additionally, it plays a significant role in establishing ten hydrophobic contacts within the active site, encompassing four Alkyl interactions, five π-Alkyl, one π-Sigma interaction with specific amino acid residues. It also has a halogen interaction with specific amino acid residues. Moreover, Trp357, Thr163, His305, Ala198, Glu233, Asp197, His101, Trp58, His299, Tyr62, Leu165, and Gln63 were the specific amino acid residues involved in the van der Waals interaction with compound **4e**.

### MD simulation

**Fig 5:**
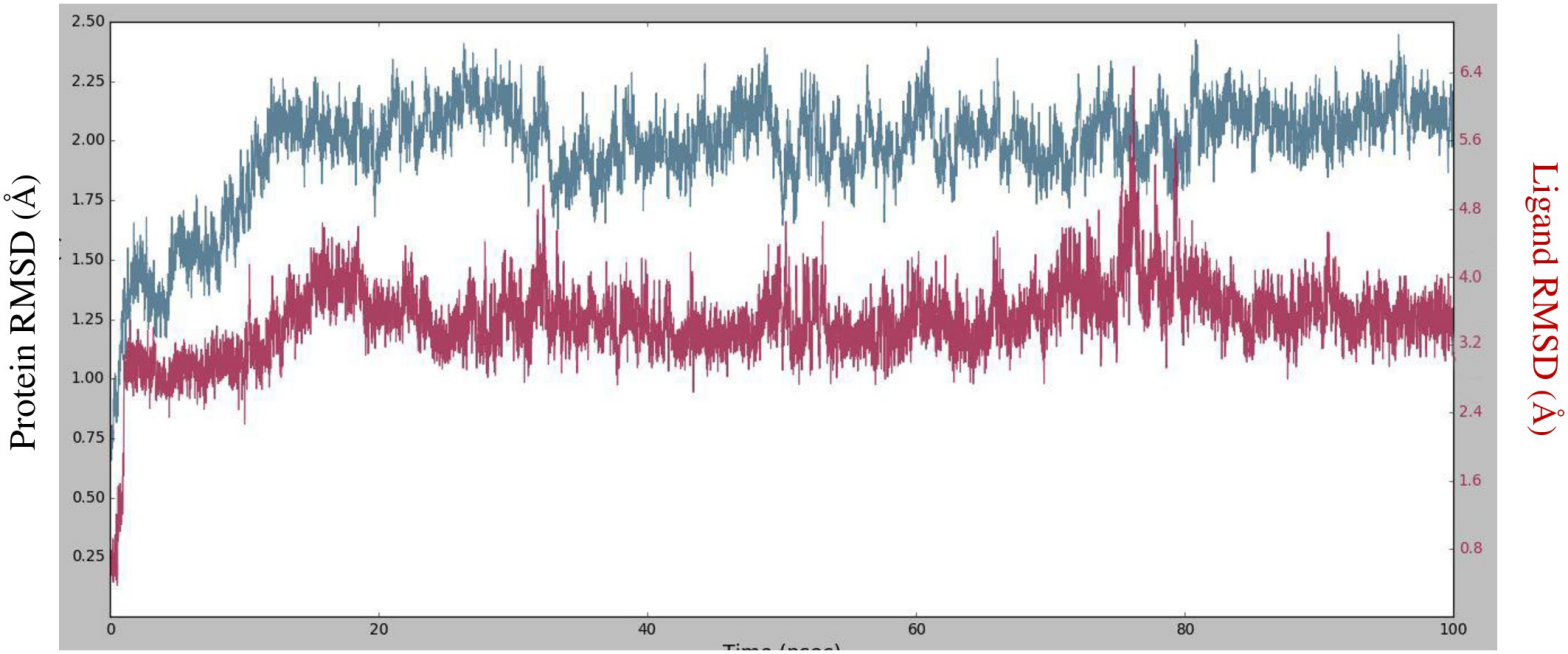
RMSD values of the protein and ligand during 100 ns MD simulation.

The results of molecular docking studies, although somewhat suggestive of the real process of protein-ligand binding, are further elucidated by MD simulations, which provide a comprehensive understanding of the interactions, including even the subtlest differences. Consequently, a MD simulation was conducted to investigate the best protein-ligand complex over a duration of 100 ns, with the aim of uncovering precise atomic-level information about molecular interactions (Fig 5). The RMSD study of the C*α* atoms revealed that the complex stabilizes after 25 nanoseconds and stays stable with slight perturbation at 80. RMSD, with respect to its initial position, rose to 2.30 for the initial 25 ns before becoming more stable thereafter. All of these RMSDs are within acceptable ranges, indicating that values remained stable in the receptor-ligand complex during the simulation.

**Fig 6:**
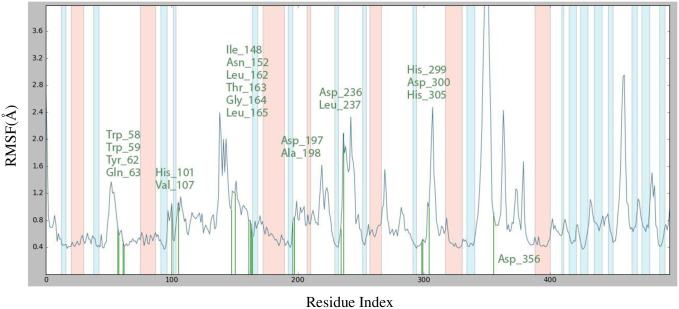
RMSF plot for C*α* of α-amylase residues in compound 4e-α-amylase complex.

The RMSF feature reveals the receptor’s average deviation from the reference position. Fig 6 depicts a complete examination of compound **4e**’s interaction with the residues of the α-amylase binding site. The residues that interact with compound **4e** are as follows: Trp58, Trp59, Tyr62, Gln63, His101, Val107, lle148, Asn152, Leu162, Thr163, Gly164, Leu165, Asp197, Ala198, Asp 236, Leu237, His299, Asp300, and His305. The values for the residues inside the binding site were determined to be less than 2 Å. The residue that interacts with compound **4e** is shown in the color green. The graphic displays the protein’s secondary structures, with helices represented by orange bands and β-strands represented by blue bands.

**Fig 7:**
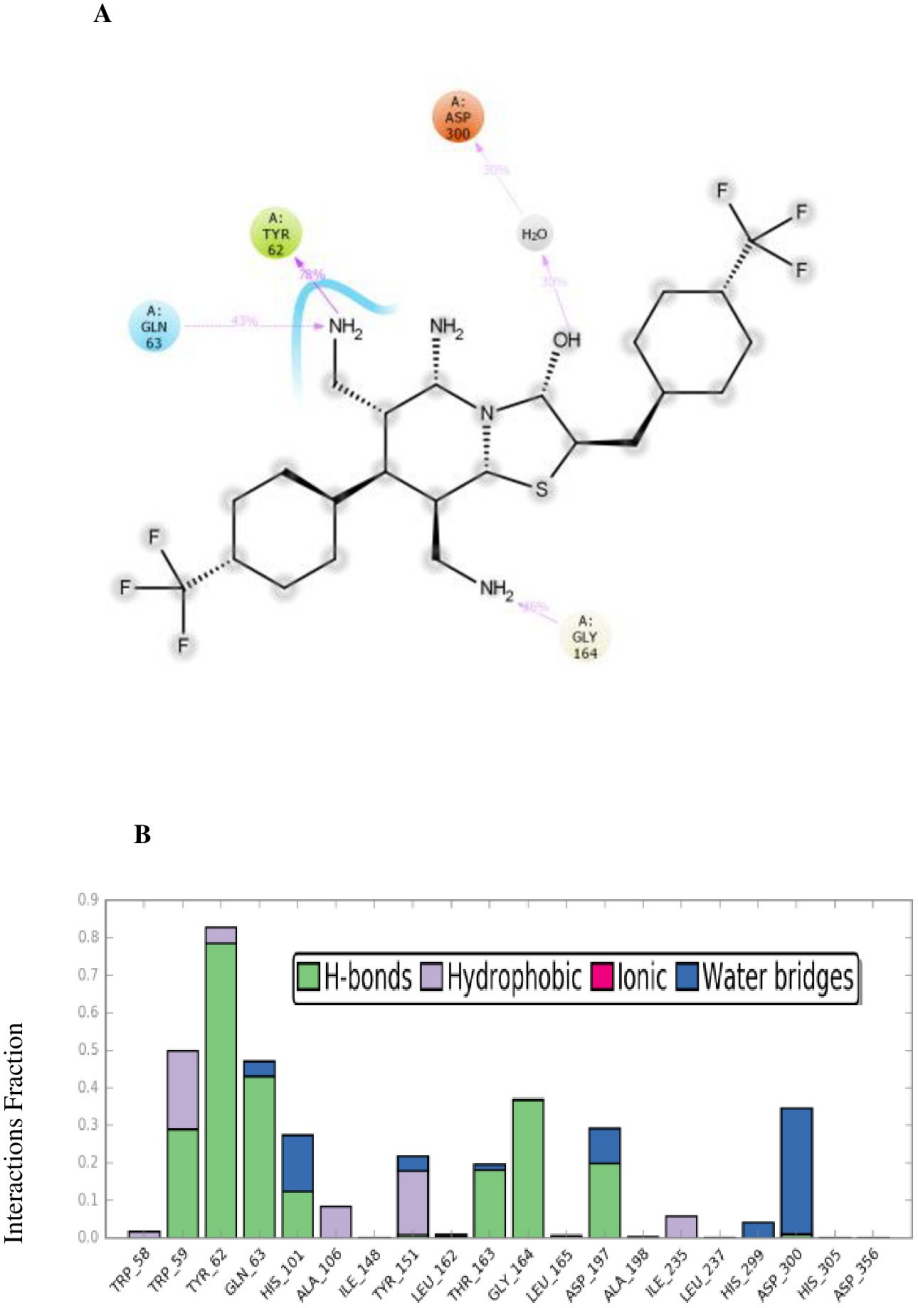
**A**: Schematic of detailed ligand atom interactions with the protein residues. **B**: Protein–ligand contacts.

During the 100 ns the compound **4e** mostly established non-covalent interactions with the amino acids Asp300, Tyr62, Gln63, and Gly164. The Fig 7 illustrate the various types of non-covalent interactions involving these amino acids, such as hydrogen bonding, hydrophobic interactions, ionic interactions, and water bridge formation. Additionally, the figures demonstrate the individual contributions of each interaction type to the overall interaction with a specific amino acid.

**Fig 8:**
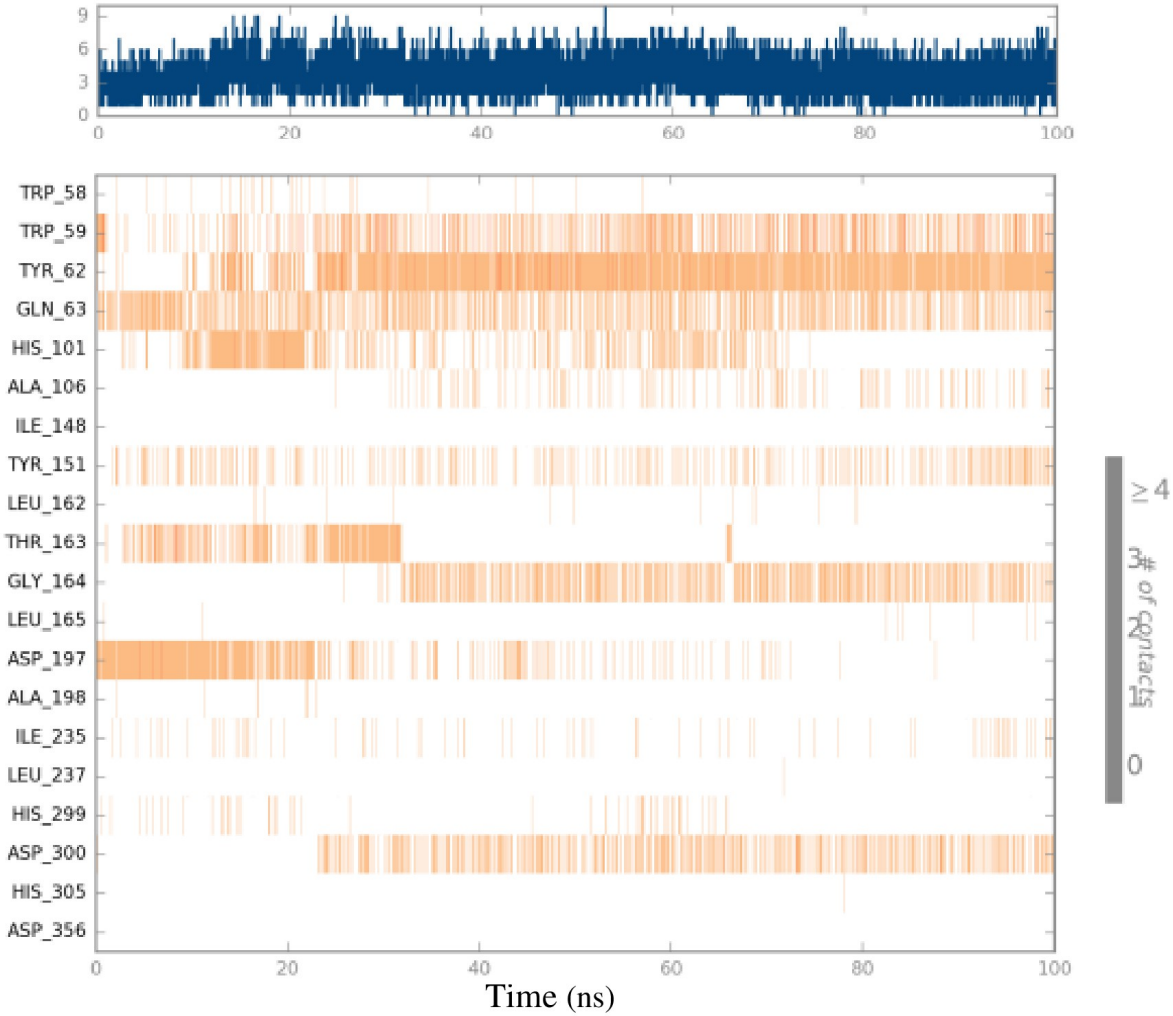
Contact points of 4e and amino acids of the α-amylase binding site during the whole simulation trajectory.

Fig 8 displays the time-dependent overall number of interactions between the ligand and protein, as well as the number of interactions specifically between the ligand and amino acids. The upper panel displays the overall number of contacts made by compound **4e** with the binding site of α-amylase along the whole journey, while the lower panel illustrates the specific interactions occurring at each residue. The residues Trp59, Tyr62, Gln63, and Tyr151 exhibited consistent interactions across all frames.

**Fig 9:**
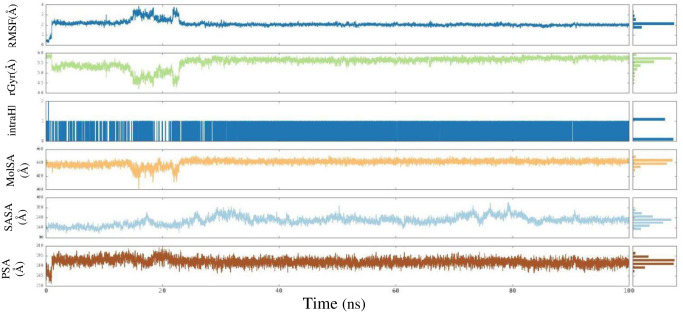
Ligand properties demonstrated by RMSD, rGyr, intraHB, MolSA SASA, PSA.

The ligand properties were examined throughout the 100-ns MD simulation and are shown in Fig 9. The RMSD of a ligand with respect to its reference conformation (t = 0) was calculated to be between 0.1 and 3.9 Å. The rGyr predicts the firmness of ligand-protein complex within the range of 4.1 to 6 Å. The presence of an intra-HB was identified. The MolSA was calculated by measuring the van der Waals surface area within the range of 419 to 431 Å. The SASA analysis measures the extent of interaction between a ligand and solvents across a 100-ns MD simulation. The resulting values range from 90 to 320 Å. The PSA, which offers insights into the solvent’s capacity to reach the surface area defined by oxygen and nitrogen atoms, The PSA value varied between 158 and 210 Å.

### In *silico* drug-likeness prediction

Drug-likeness prediction is important in drug discovery and development because it allows researchers to choose compounds for further investigation while also reducing the time and expenses associated with the drug development process. The procedure entails analyzing many physical and chemical characteristics of a molecule, including its molecular weight, lipophilicity, hydrogen bonding capability, and solubility. Lipinski’s rule, popularly known as the "Ro5," is a widely used approach for estimating the suitability of a molecule as a drug. According to this rule, compounds that fail to meet certain requirements are more likely to have inadequate absorption or penetration. These criteria include having more than 5 HBD combining NH and OH groups, a molecular weight exceeding 500, a Log P value above 5, and more than 10 HBA including N and O atoms [18]. We examined all of our derivatives using the SwissADME [19] online web server. Our analysis revealed that all thiazolo[3,2-*a*] pyridine-6,8-dicarbonitrile derivatives adhere to the Ro5, meaning that they do not have more than one violation. Consequently, there should be no concerns regarding their oral bioavailability. The pharmacokinetic parameters indicated that the studied compounds **4a**, **4d**, **4f**, and **4j** are rapidly absorbed *via* the GI after oral intake. Other metrics, such as molecular weight, HBD, HBA, and rotatable bonds, are within our synthesized compounds’ range, suggesting that they might easily traverse cell membranes. The Brenk and PAINS structural warnings have been utilized in pharmaceutical chemistry to determine whether sections of the structure are unstable, reactive, and dangerous [20, 21]. Except for compound **4b, 4h**, which contained two nitro_groups and an oxygen-nitrogen_single_bond, all of the compounds had adequate Brenk descriptions. Furthermore, with the exception of compound **4j**, which had an anil_di_alk_B warning, all of the compounds had strong PAINS descriptions, indicating that they might be potential drug prospects. The SA score is a measure used to assess the ease of synthesizing drug-like molecules; all of the compounds have a favorable SA value, suggesting that they can be easily synthesized, as shown in Table 4.

**Table 4:** Physicochemical, pharmacokinetics, and medicinal chemistry properties of the compounds (4a–j).

	MW (g/mol)	HBA	HBD	NRB	Consensus Log Po/w *	MR	GI Absorption	TPSA (Å^2^)	Lipinski	Pfizer	PAINS (alert)	Brenk	Bioavailability Score	Synthetic accessibility score
**4a**	451.33	3	1	2	3.91	118.77	High	123.84	Yes	Yes	0	0	0.55	4.57
**4b**	472.43	7	1	4	1.40	126.39	Low	215.48	Yes**	Yes	0	*****2	0.55	4.74
**4c**	520.22	3	1	2	4.96	128.79	Low	123.84	Yes***	Yes	0	0	0.55	4.65
**4d**	418.42	5	1	2	3.48	108.66	High	123.84	Yes	Yes	0	0	0.55	4.55
**4e**	518.43	9	1	4	4.93	118.75	Low	123.84	Yes***	Yes	0	0	0.55	4.80
**4f**	451.33	3	1	2	3.89	118.77	High	123.84	Yes	Yes	0	0	0.55	4.65
**4g**	414.44	5	3	2	2.04	112.79	Low	164.30	Yes	Yes	0	0	0.55	4.60
**4h**	472.43	7	1	4	1.39	126.39	Low	215.48	Yes**	Yes	0	*****2	0.55	4.68
**4i**	474.49	7	3	4	2.15	125.78	Low	182.76	Yes	Yes	0	0	0.55	4.89
**4j**	468.57	3	1	4	2.88	137.16	High	130.32	Yes	Yes	****1	0	0.55	5.12

* Average of five prediction,

**1 violation: NorO>10,

***1 violation: MW>500,

****1 alert: anil_di_alk_B,

*****2 alerts: nitro_group, oxygen-nitrogen_single_bond

### In *silico* ADMET properties

ADMET properties play a crucial role in the early stages of drug discovery and development. Computational models have been developed to evaluate ADMET features, such as absorption, distribution, metabolism, and toxicity, for the purpose of drug design and lead optimisation. These models use ADMET lab 2.0 [22]. This integrated online platform offers a reliable and extensive means to accurately predict and assess ADMET properties. The considered ADMET profile includes various factors such as physicochemical properties, BBB permeability, Caco-2 permeability, VD, PGP substrate, HIA, MDCK permeability, CL, T_1/2_, potential for eye corrosion, eye irritation, respiratory toxicity, AMES toxicity, carcinogenicity, and synthetic accessibility score. CaCo-2 cells, which are derived from human colon epithelial cells, are widely utilized as a model system to assess the ability of drugs to be absorbed in the human intestines. In contrast, MDCK cells, known for their shorter growth cycle compared to CaCo-2 cells, are valuable for evaluating the rapid permeability of drug molecules [23]. The obtained results of CaCo-2 cell permeability for our synthesized compounds demonstrated values within a desirable range, indicating that these compounds exhibit excellent intestinal absorption capabilities. Furthermore, all the compounds exhibited favourable MDCK cell permeability, indicating a higher likelihood of elimination through kidney cells. The results also indicated that all the compounds, except compound **4j**, displayed characteristics that suggest they are substrates for PGP. To elaborate further, compounds **4b**, **4c**, **4f**, **4g**, **4h**, and **4i** have been identified as inhibitors of PGP, whereas compounds **4a**, **4d**, **4e**, and **4j** do not exhibit inhibitory effects on PGP. Considering the HIA values, it can be concluded that all the compounds possess a favourable probability of being absorbed through the intestinal membrane. The predictions for efflux values indicate that all compounds have the potential to penetrate the BBB, and among them, compound **4i** exhibits the lowest BBB value. The involvement of the OH in hydrogen bonding is evident, as it ultimately results in a decrease in the compound’s ability to penetrate the BBB. These findings align well with the study conducted by Young *et al*., which demonstrated that excessive hydrogen bonding limits the penetration of antihistamines into the CNS [24]. The percentage representation of the plasma protein binding model indicates the degree to which a compound strongly binds to carrier proteins in the blood. The plasma protein binding values for all the compounds surpass 94%, indicating that our synthesized thiazolo[3,2-*a*] pyridine-6,8-dicarbonitrile derivatives possess ample bioavailability and are unlikely to demonstrate significant binding to carrier proteins in the blood. Cytochrome P450 is a heme-containing enzyme that is found on the lipid bilayer of the endoplasmic reticulum of hepatocytes, where the majority of drug, steroid, and carcinogen metabolism occurs [25]. CYP P450 has two main subtypes: CYP2D6 and CYP3A4. The findings demonstrated negative inhibitory capacity for CYP2D6 enzymes, indicating that it is safe for pharmacokinetic interactions. Regarding carcinogenicity, compounds **4b**, **4h**, and **4j** were determined to be non-carcinogenic, while compounds **4d** and **4g** do not exhibit a clear classification as either carcinogenic or non-carcinogenic. On the other hand, the other compounds have a likelihood of being carcinogenic. The AMES toxicity assessment revealed that compounds **4b**, **4d**, **4g**, **4h**, and **4j** are non-AMES toxic, while compounds **4c** and **4f** do not exhibit a clear classification as either AMES or non-AMES toxic. However, the other compounds have a probability of being toxic. Overall, all the compounds demonstrated a favourable ADMET profile. All values are tabulated in Table 5.

**Table 5:** ADMET profile of the compounds (4a–j).

Absorption and Distribution												
	Caco-2 Permeability	MDCK Permeability (nm/s)	PGP-Inhibitor	PGP-Substrate	HIA	PPB	VD	BBB penetration (c.brain/ c.blood)				
**4a**	-4.685	172	++0.812	---0.05	---0.004	101.12	0.276	--0.148				
**4b**	-4.648	3980	---0.015	---0.013	---0.007	100.77	0.633	-0.343				
**4c**	-4.751	224	--0.22	---0.004	---0.007	102.15	0.718	---0.077				
**4d**	-4.732	290	+++0.945	--0.104	---0.007	100.66	0.346	+0.152				
**4e**	-5.030	214	+++0.957	---0.073	---0.004	100.86	0.742	--0.123				
**4f**	-4.628	410	--0.126	---0.006	---0.005	101.06	0.299	--0.132				
**4g**	-4.973	100	---0.003	--0.221	---0.006	99.96	0.348	---0.065				
**4h**	-4.769	4170	--0.176	---0.068	---0.008	100.82	0.698	--0.216				
**4i**	-4.911	134	---0.013	-0.034	---0.028	100.45	0.312	---0.005				
**4j**	-4.744	290	+++0.995	++0.838	--0.288	99.59	0.619	--0.297				
Metabolism											Elimination	
	CYP1A2 inhibitor	CYP1A2 Substrate	CYP2C19 inhibitor	CYP2C19 Substrate	CYP2C9 inhibitor	CYP2C9 Substrate	CYP2D6 inhibitor	CYP2D6 Substrate	CYP3A4 inhibitor	CYP3A4 Substrate	T_1/2_	CL
**4a**	+0.648	--0.168	++0.875	--0.196	+++0.906	---0.064	-0.347	---0.057	-0.484	+++0.931	0.033	6.435
**4b**	-0.389	---0.077	++0.731	--0.209	+++0.941	--0.234	---0.019	-0.088	++0.886	+++0.905	0.086	5.658
**4c**	+0.530	-0.410	+++0.905	-0.385	+++0.952	---0.094	---0.039	---0.061	+0.550	+++0.937	0.013	6.559
**4d**	+0.593	--0.116	++0.718	--0.187	+++0.901	---0.100	---0.026	---0.092	-0.451	+0.913	0.023	6.747
**4e**	--0.286	--0.219	++0.837	--0.136	+++0.947	--0.114	---0.005	---0.084	+0.508	++0.875	0.007	7.580
**4f**	++0.631	--0.185	++0.891	-0.409	+++0.955	--0.105	---0.009	---0.069	++0.701	+++0.932	0.036	6.543
**4g**	++0.74	---0.063	+0.663	---0.066	++0.863	++0.817	---0.030	---0.090	++0.818	++0.838	0.475	7.532
**4h**	--0.241	---0.052	+0.551	--0.107	++0.725	--0.256	---0.006	---0.096	+0.544	++0.868	0.063	5.338
**4i**	-0.371	++0.801	+0.695	++0.742	+++0.916	++0.786	---0.023	--0.143	++0.833	+++0.923	0.287	6.479
**4j**	-0.499	+0.558	+0.668	+0.658	++0.856	---0.057	---0.019	--0.144	+0.666	+++0.922	0.078	6.752
Toxicity												
	AMES toxicity	Carcinogenicity	Eye corrosion	Eye irritation	hERG	H-HT	LD_50_	Respiratory toxicity				
**4a**	++0.879	--0.182	---0.003	---0.014	---0.003	+++0.919	830.81	--0.219				
**4b**	+++0.943	++0.793	---0.003	--0.153	---0.004	+++0.959	811.612	+0.57				
**4c**	+0.515	---0.219	---0.003	---0.067	---0.002	+++0.971	815.068	--0.289				
**4d**	+++0.91	-0.355	---0.003	---0.01	---0.002	+++0.957	936.744	--0.264				
**4e**	--0.126	---0.096	---0.003	---0.024	---0.003	+++0.975	169.317	++0.715				
**4f**	+0.695	--0.263	---0.003	---0.037	---0.003	+++0.959	791.593	-0.359				
**4g**	++0.814	-0.366	---0.003	--0.158	---0.001	++0.734	773.514	---0.085				
**4h**	+++0.987	++0.809	---0.003	---0.048	---0.007	+++0.951	718.371	++0.757				
**4i**	--0.225	--0.132	---0.003	--0.215	---0	+++0.915	648.985	--0.201				
**4j**	+++0.968	++0.779	---0.003	---0.044	---0.005	+++0.911	538.009	++0.813				

## 3. Conclusion

A series of thiazolo[3,2-a] pyridine-6,8-dicarbonitrile derivatives were prepared based on the reaction of thioglycolic acid, malononitrile, and aromatic aldehyde derivatives in absolute EtOH at reflux conditions, and all of the compounds were investigated for in *silico* study. During the docking, we successfully docked all of the compounds with the enzyme α-amylase. Our results indicate that all of the compounds have many potential inhibitors. Notably, compound **4e** exhibited a strong binding affinity with the α-amylase. The stability, RMSD, and RMSF values of the ligand-protein complex for **4e** were assessed by MD simulation, and outcome of docking was confirmed using MD simulation. The pharmacokinetic and drug-like properties of thiazolo[3,2-*a*] pyridine-6,8-dicarbonitrile derivatives are valuable and provide an informative and promising investigation as effective α-amylase inhibitors. The findings of the chemoinformatics study would need to be validated by more in vivo and in vitro research.

## 4. Experimental

### General

All compounds were obtained from Merck and Aldrich chemical companies. The melting points were measured using an Electro-thermal 9100. The FT-IR spectra were taken using KBr discs on a Bruker tensor 27 spectrometer, with absorbencies reported in cm^−1^. The NMR spectra were recorded with a Bruker DRX-400 AVANCE instrument (400 MHz for ^1^H and 100 MHz for ^13^C) with deuterated dimethyl sulfoxide (DMSO-*d*_*6*_) as solvent and tetramethylsilane (TMS) as an internal standard. Chemical shifts are given in ppm (*δ*) and coupling constant (*J*) is reported in Hertz (Hz).

### General method to synthesis thiazolo[3,2-*a*]pyridine-6,8-dicarbonitrile (4a–j)

A mixture of thioglycolic acid **1** (1 mmol), malononitrile **2** (2 mmol) aldehydes **3** (2 mmol) in the presence of piperidine (50 mol%) was heated in EtOH (5 mL) at reflux condition. After appropriate time, yellow precipitate was formed (monitored by TLC). After confirming the completion of the reaction using TLC, the solid product was filtered and washed to get the pure product.

### Selected spectral data

***(Z)-5-amino-2-(4-chlorobenzylidene)-7-(4-chlorophenyl)-3-oxo-3*,*7-dihydro-2H-thiazolo[3*,*2-a]pyridine-6*,*8-dicarbonitrile* (4a):** Yellow solid; yield: (56%); mp: 265–267°C; IR (KBr) (ν_max_ /cm^-1^): 3378, 3282, 2202, 1717, 1654, 1560, 1409, 1333, 1155, 1092, 772; ^1^H NMR (400 MHz, DMSO-*d*_*6*_): *δ* 4.58 (1H, s, CH), 7.37 (4H, br, Ar-H), 7.49 (2H, s, NH_2_), 7.55 (2H, d, *J* = 6.0 Hz, ArH), 7.59 (2H, d, *J* = 9.0 Hz, ArH), 7.76 (1H, s, CH); ^13^C{^1^H} NMR (100 MHz, DMSO*-d*_*6*_): *δ* 40.2 (CH), 63.2 (**C**CN), 87.3 (**C**CN), 115.7 (CN), 118.5 (CN), 119.5 (CH = **C**-S), 128.8, 129.6, 130.0 (Ar), 130.4 (C-Cl), 131.6 (**C**H = C-S), 131.8, 132.7 (Ar), 135.1 (C-Cl), 140.7 (= CH), 143.0 (**C** = C-CN), 147.9 (NC = O), 165.2 (= C-NH_2_).

***(Z)-5-amino-2-(2-nitrobenzylidene)-7-(2-nitrophenyl)-3-oxo-3*,*7-dihydro-2H-thiazolo[3*,*2-a]pyridine-6*,*8-dicarbonitrile* (4b):** Light brown solid; yield: (46%); mp: 236–238°C; IR (KBr) (ν_max_ /cm^-1^): 3393, 3299, 2199, 1721, 1654, 1563, 1524, 1412, 1333, 1258, 1169, 867, 781; ^1^H NMR (400 MHz, DMSO-*d*_*6*_): *δ* 5.08 (1H, s, CH), 7.52–7.89 (8H, m, Ar-H), 8.00 (1H, s, CH_vinyl_), 8.14 (2H, s, NH_2_); ^13^C{^1^H} NMR (100 MHz, DMSO-*d*_*6*_): *δ* 36.1 (CH), 62.5 (**C**CN), 86.5 (**C**CN), 115.3 (CN), 118.1 (CN), 123.3 (CH = **C**-S), 124.2, 125.7, 128.3, 128.6, 128.8, 129.7, 131.3, 132.0, 134.0, 135.0, 143.9 (**C** = C-CN), 147.7 (C-NO_2_), 148.2 (NC = O), 148.5 (C-NO_2_), 164.4 (= C-NH_2_).

***(Z)-5-amino-2-(2*,*6-dichlorobenzylidene)-7-(2*,*6-dichlorophenyl)-3-oxo-3*,*7-dihydro-2H-thiazolo[3*,*2-a]pyridine-6*,*8-dicarbonitrile* (4c):** Yellow solid; yield: (44%); mp: 299–301°C; IR (KBr) (ν_max_ /cm^-1^): 3420, 3330, 2200, 1719, 1650, 1562, 1422, 1333, 1257, 1155, 856, 778; ^1^H NMR (400 MHz, DMSO-*d*_*6*_): *δ* 5.46 (1H, s, CH), 7.31–7.57 (6H, m, Ar-H and 2H, s, NH_2_), 7.76 (1H, s, CH); ^13^C{^1^H} NMR (100 MHz, DMSO-*d*_*6*_): *δ* 37.5 (CH), 59.9 (**C**CN), 84.9 (**C**CN), 114.8 (CN), 117.7 (CN), 126.8 (CH = **C**-S), 128.1, 128.9, 128.9, 130.6, 130.9, 131.1, 132.2, 132.4, 133.0, 134.9, 135.6, 143.6 (**C** = C-CN), 149.0 (NC = O), 163.4 (= C-NH_2_).

***(Z)-5-amino-2-(4-fluorobenzylidene)-7-(4-fluorophenyl)-3-oxo-3*,*7-dihydro-2H-thiazolo[3*,*2-a]pyridine-6*,*8-dicarbonitrile* (4d):** Yellow solid; yield: (35%); mp: 267–269°C; IR (KBr) (ν_max_ /cm^-1^): 3381, 3283, 2199, 1721, 1656, 1604, 1561, 1507, 1414, 1335, 1234, 1155, 827; ^1^H NMR (400 MHz, DMSO-*d*_*6*_): *δ* 4.56 (1H, s, CH), 7.11–7.17 (2H, m, Ar-H), 7.29–7.37 (4H, m, Ar-H), 7.46 (2H, br s, Ar-H), 7.64 (2H, s, NH_2_), 7.77 (1H, s, CH); ^13^C{^1^H} NMR (100 MHz, DMSO-*d*_*6*_): *δ* 40.1 (CH), 63.5 (**C**CN), 87.5 (**C**CN), 115.5 (CN), 115.7 (Ar), 115.8 (Ar), 116.6 (CN), 116.8 (CH = **C**-S), 118.4, 118.4, 118.6, 129.4, 129.5, 130.2, 130.2, 130.6, 132.6, 132.7, 138.0, 138.0, 142.8 (**C** = C-CN), 147.8 (NC = O), 160.5, 161.6, 162.9, 164.1, 165.3 (= C-NH_2_).

***(Z)-5-amino-2-(2-chlorobenzylidene)-7-(2-chlorophenyl)-3-oxo-3*,*7-dihydro-2H-thiazolo[3*,*2-a]pyridine-6*,*8-dicarbonitrile* (4f):** Green solid; yield: (30%); mp: 296–298°C; IR (KBr) (ν_max_ /cm^-1^): 3388, 3285, 2199, 1716, 1651, 1561, 1410, 1332, 1152, 1044, 755; ^1^H NMR (400 MHz, DMSO-*d*_*6*_): *δ* 4.99 (1H, s, CH), 7.25–7.60 (8H, m, Ar-H and 2H, s, NH_2_), 7.87 (1H, s, CH); ^13^C{^1^H} NMR (100 MHz, DMSO-*d*_*6*_): *δ* 38.2 (CH), 62.3 (**C**CN), 86.8 (**C**CN), 115.4 (CN), 118.2 (CN), 122.3 (CH = **C**-S), 126.5, 128.1, 128.4, 128.9, 129.7, 129.9, 130.5, 130.5, 131.4, 132.1, 132.2, 134.3, 138.0, 143.4 (**C** = C-CN), 148.2 (NC = O), 164.8 (= C-NH_2_).

***(Z)-5-amino-2-(4-nitrobenzylidene)-7-(4-nitrophenyl)-3-oxo-3*,*7-dihydro-2H-thiazolo[3*,*2-a]pyridine-6*,*8-dicarbonitrile* (4h):** Brown solid; yield: (53%); mp: 240–242°C; IR (KBr) (ν_max_ /cm^-1^): 3374, 3272, 2203, 1719, 1650, 1566, 1516, 1414, 1341, 1158, 845, 708; ^1^H NMR (400 MHz, DMSO-*d*_*6*_): *δ* 4.82 (1H, s, CH), 7.57 (2H, s, NH_2_), 7.65 (2H, d, Ar-H, *J* = 9.0 Hz), 7.83 (2H, d, Ar-H, *J* = 9.0 Hz), 7.89 (1H, s, CH), 8.19 (2H, d, Ar-H, *J* = 9.0 Hz), 8.28 (2H, d, Ar-H, *J* = 9.0 Hz); ^13^C{^1^H} NMR (100 MHz, DMSO-*d*_*6*_): *δ* 40.4 (CH), 62.5 (**C**CN), 87.1 (**C**CN), 115.5 (CN), 118.3 (CN), 123.2 (CH = **C**-S), 124.1, 124.5 129.1, 129.6, 131.1, 138.9, 143.5 (**C** = C-CN), 147.3, 147.4, 148.1, 148.7 (NC = O), 164.9 (= C-NH_2_).

### Computational studies

#### Molecular docking

The α-amylase enzyme’s interaction with synthesized compounds was studied using Schrodinger’s Maestro Molecular Modelling platform. The protein 3D structure was obtained from PDB (4W93) [16]. Protein was prepared using the Protein Preparation Wizard [26], and missing residues were modified. Using the ligprep module, compounds were optimized. The OPLS_2005 force field was used to prepare the ligand at pH 7.0± 2 [27]. With standard accuracy and flexible ligand sampling, Glide makes 26-A grid boxes at each binding site and reports 10 poses for each ligand.

#### Molecular dynamic simulation

Desmond was used for MD simulation via Schrödinger-Maestro [28]. The MD simulation conducted on the complex after the docking process produced the results. The SPC model cells exhibited an orthorhombic structure and were completely filled by water. The charge of the complex was equilibrated by introducing a sufficient quantity of Na ions. The simulation had a duration of 100 nanoseconds. The NPT ensemble was used to maintain a fixed number of atoms, a pressure of 1.01325 bar, and a temperature of 300 K. The first thermostat and barostat used in the system were the 1.0‐ps Nose-Hoover chain and 2.0‐ps Martyna-Tobias-Klein techniques, respectively. The MD simulation was assessed using the maestro simulation interaction diagram.

## Supporting information

S1 File The supporting information is available and provided copies of IR, ^1^H NMR and ^13^C NMR spectra for all products. (DOCX)

## Abbreviations

2D: Two-dimensional
3D: Three dimensional
Å: Angstroms
ADMET: Absorption, Distribution, Metabolism Excretion
BBB: Blood-brain barrier
CaCo-2: Human colon epithelial cancer cell line
CL: Clearance
CNS: Central nervous system
DMSO: Dimethyl sulfoxide
Et_3_N: Triethylamine
EtOH: Ethanol
FT-IR: Fourier transform infrared spectroscopy
GI: Gastrointestinal tract
HBA: Hydrogen bond acceptors
HBD: Hydrogen bond donors
HCl: Hydrochloric acid
hERG: Human ether-a-go-go-related gene
H-HT: Human hepatotoxicity
HIA: Human intestinal absorption
intra-HB: Intramolecular hydrogen bond
LD50: Median lethal dose
MD: Molecular dynamic
MDCK: Madin darby canine kidney
MHz: Megahertz
MolSA: Molecular surface area
MR: Molar refractivity
MW: Molecular weight
NMR: Nuclear magnetic resonance
NRB: Number of rotatable bonds
OH: Hydroxyl group
PAINS: Pan assay interference substances
PDB: Plasma protein binding
PGP: P-Glycoprotein
P-M: Poor-moderate
PSA: Polar surface area
rGyr: Radius of gyration
RMSD: Root-Mean-Square Deviation
RMSF: Root Mean Square Fluctuation
Ro5: Lipinski’s Rule of five
SA: Synthetic accessibility
SASA: Solvent accessible surface area
T_1/2_: Half-life
T2DM: Type 2 diabetes mellitus
TLC: Thin layer chromatography
TMS: Tetramethylsilane
TPSA: Topological polar surface area
VD: Volume of distribution
α-amylase: Alpha-amylase
