# Supplementary material for: Synthesis, molecular docking study, MD simulation, ADMET, and drug likeness of new thiazolo[3,2-a]pyridine-6,8-dicarbonitrile derivatives as potential anti-diabetic agents
Source: PLoS One. 2024 Sep 12;19(9):e0306973. doi: 10.1371/journal.pone.0306973 (PMC11392235; doi:10.1371/journal.pone.0306973)
Supplement: S1 File — (DOCX) [file pone.0306973.s001.docx]

**Synthesis, Molecular Docking Study, MD Simulation, ADMET, and Drug Likeness of New Thiazolo[3,2- *a*]pyridine-6,8-dicarbonitrile Derivatives as Potential Anti-Diabetic Agents**

Fatemeh Aghahosseini^a^, Mohammad Bayat^a^*, Zahra Sadeghian^a^, Davood Gheidari^b^, Fatemeh Safari^b^

*^a^Department of Chemistry, Faculty of Science, Imam Khomeini International University, Qazvin, Iran*

*^b^Department of Chemistry, Faculty of Science, University of Guilan, Rasht, Iran*

**Contents**

[Figure 1. IR spectra of compound **4a** 2](#_Toc87885915)

[Figure 2. ^1^HNMR spectra of compound **4a**  3](#_Toc87885916)

[Figure 3. ^13^CNMR spectra of compound **4a** 4](#_Toc87885918)

[Figure 4. IR spectra of compound **4b** 5](#_Toc87885919)

[Figure 5. ^1^HNMR spectra of compound **4b** 6](#_Toc87885922)

[Figure 6. ^13^CNMR spectra of compound **4b** 7](#_Toc87885923)

[Figure 7. IR spectra of compound **4c** 8](#_Toc87885924)

[Figure 8. ^1^HNMR spectra of compound **4c** 9](#_Toc87885925)

[Figure 9. ^13^CNMR spectra of compound **4c** 1](#_Toc87885926)0

[Figure 10. IR spectra of compound **4d** 11](#_Toc87885927)

[Figure 11. ^1^HNMR spectra of compound **4d** 12](#_Toc87885929)

[Figure 12. ^13^CNMR spectra of compound **4d** 13](#_Toc87885930)

[Figure 13. IR spectra of compound **4e** 14](#_Toc87885931)

[Figure 14. ^1^HNMR spectra of compound **4e** 15](#_Toc87885932)

[Figure 15. ^13^CNMR spectra of compound **4e** 16](#_Toc87885933)

[Figure 16. IR spectra of compound **4f** 17](#_Toc87885934)

[Figure 17. ^1^HNMR spectra of compound **4f** 18](#_Toc87885935)

[Figure 18. ^13^CNMR spectra of compound **4f** 19](#_Toc87885936)


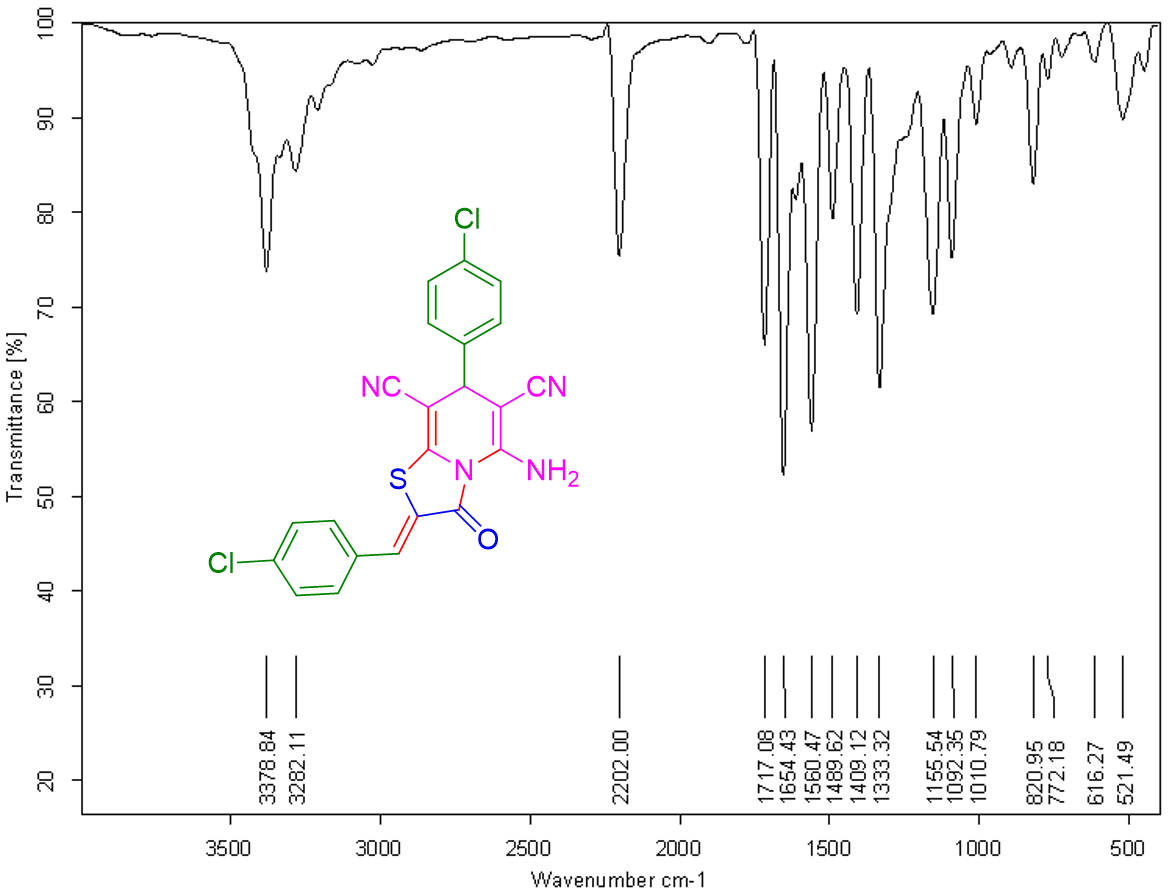


Figure 1. IR spectra of compound **4a**


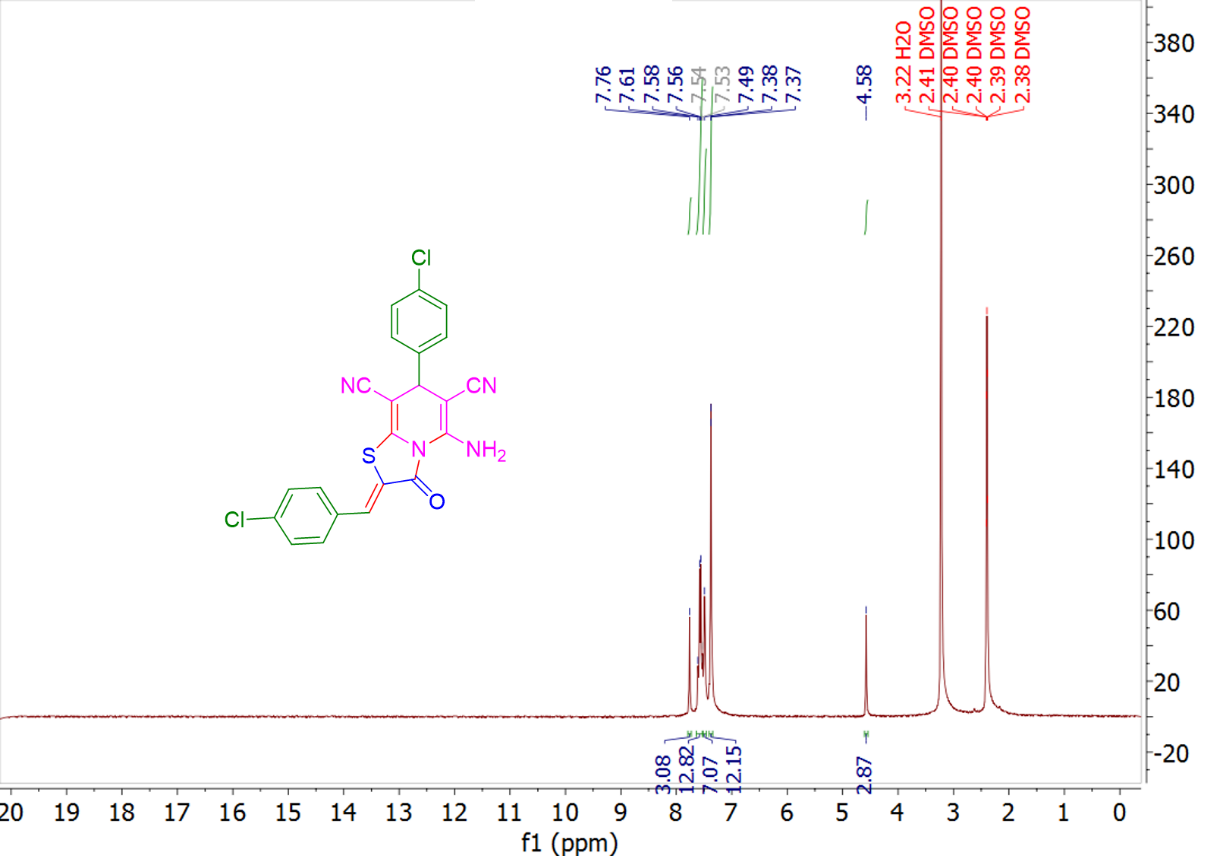


Figure 2. ^1^HNMR spectra of compound **4a**


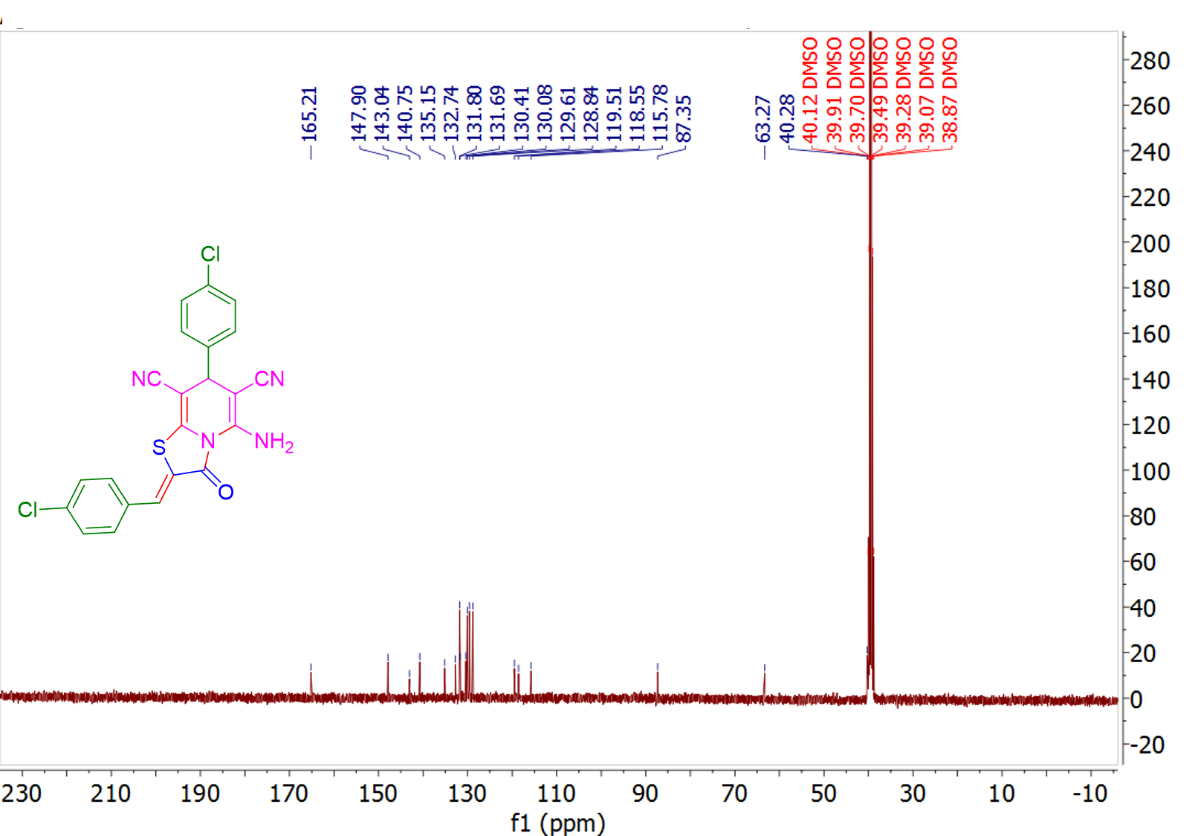


Figure 3. ^13^CNMR spectra of compound **4a**


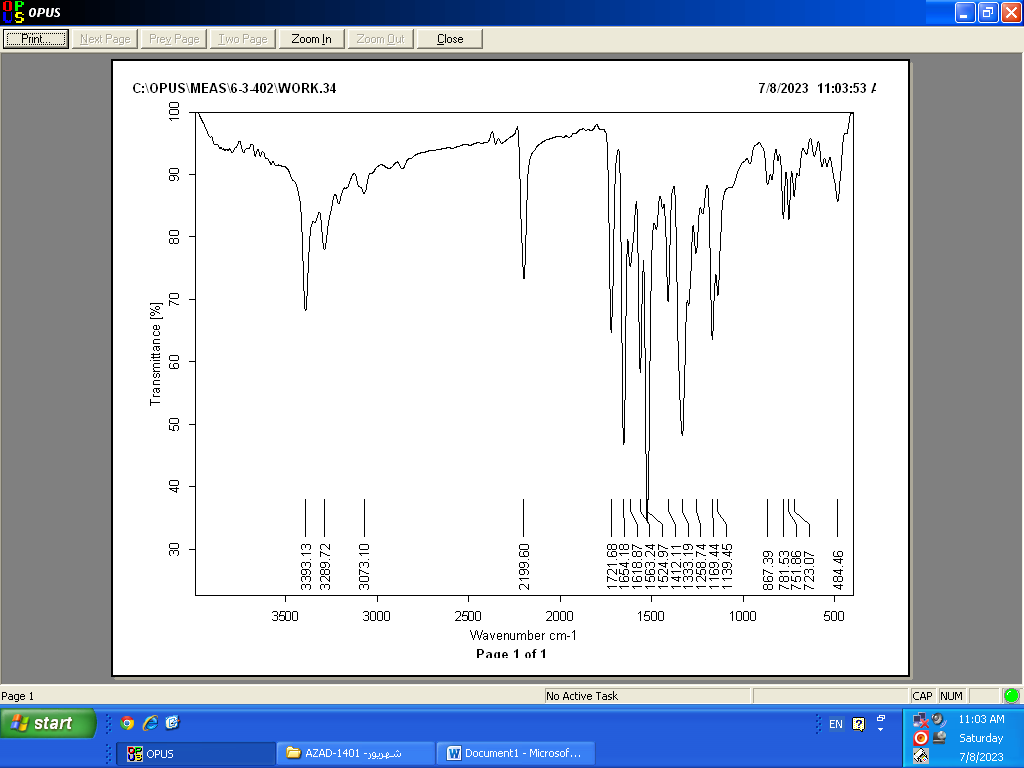


Figure 4. IR spectra of compound **4b**


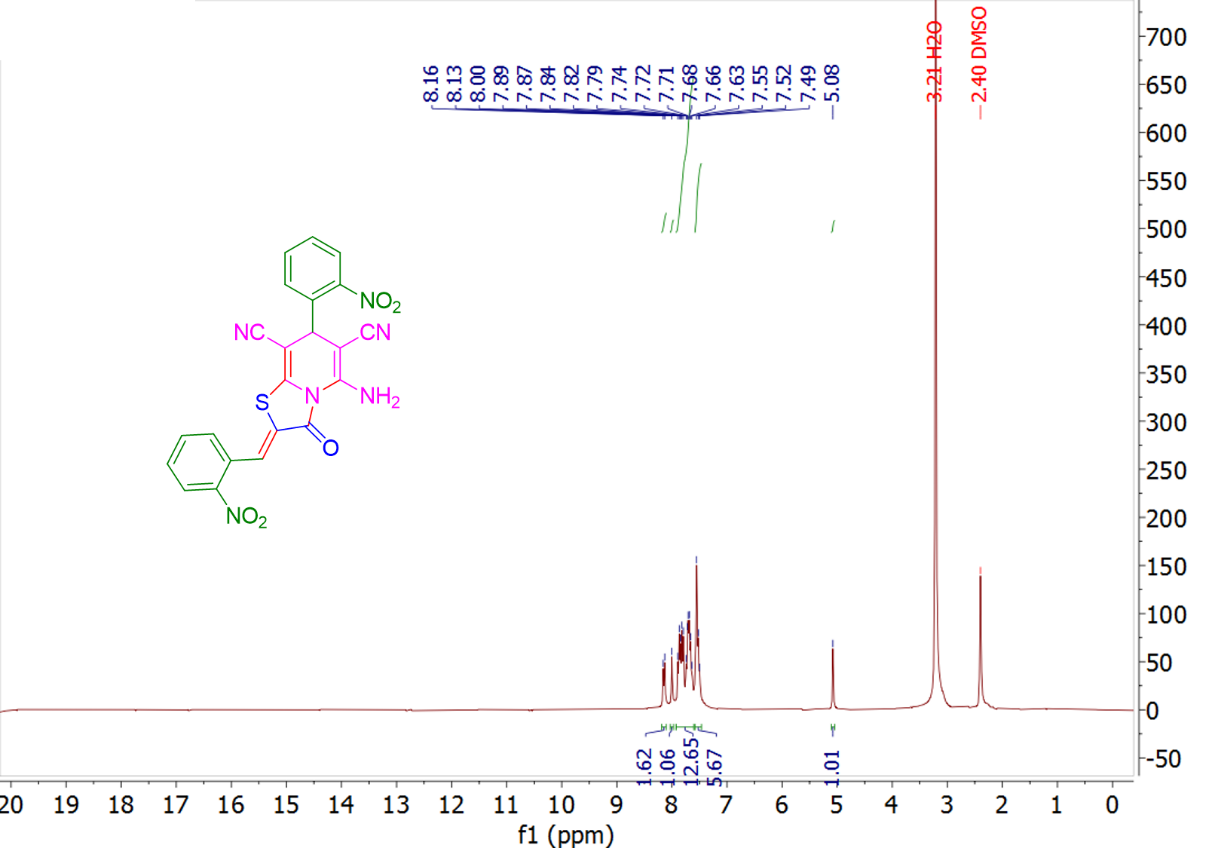


Figure 5. ^1^HNMR spectra of compound **4b**


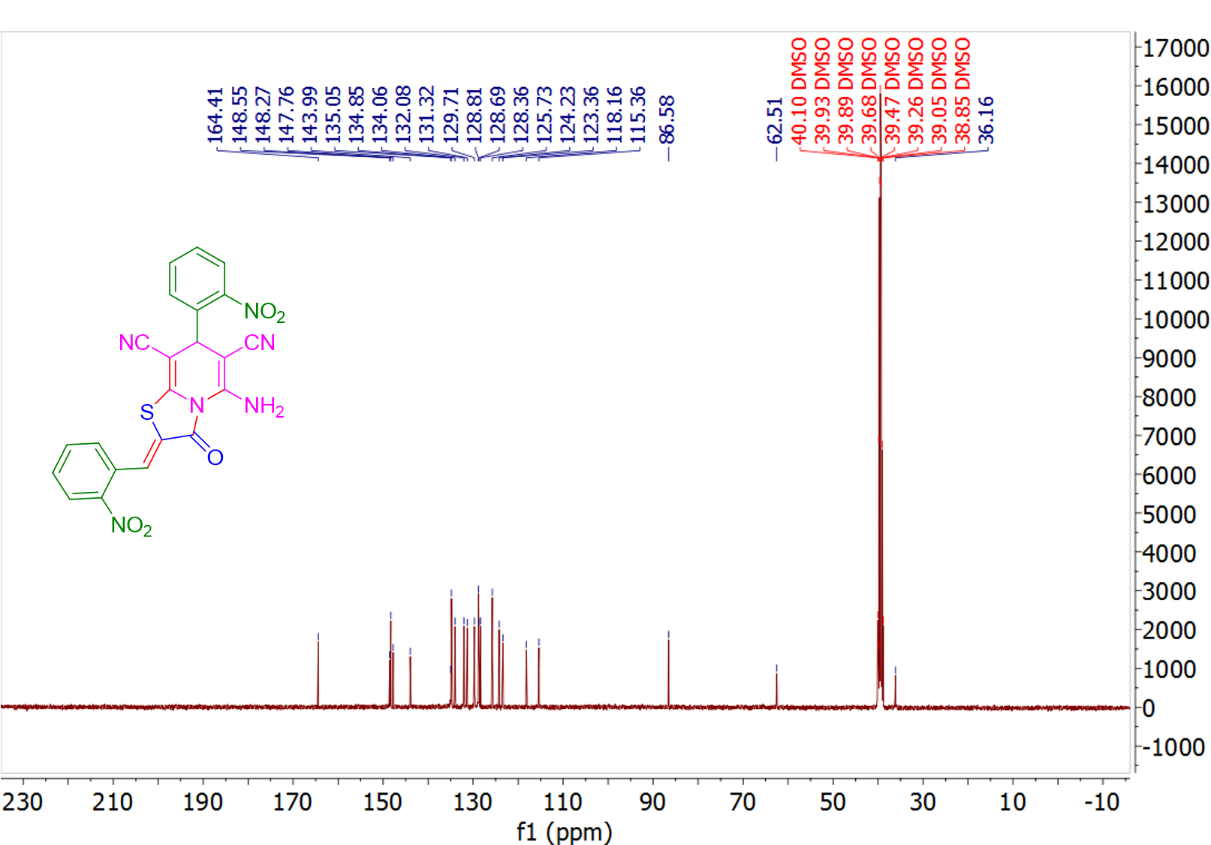


Figure 6. ^13^CNMR spectra of compound **4b**


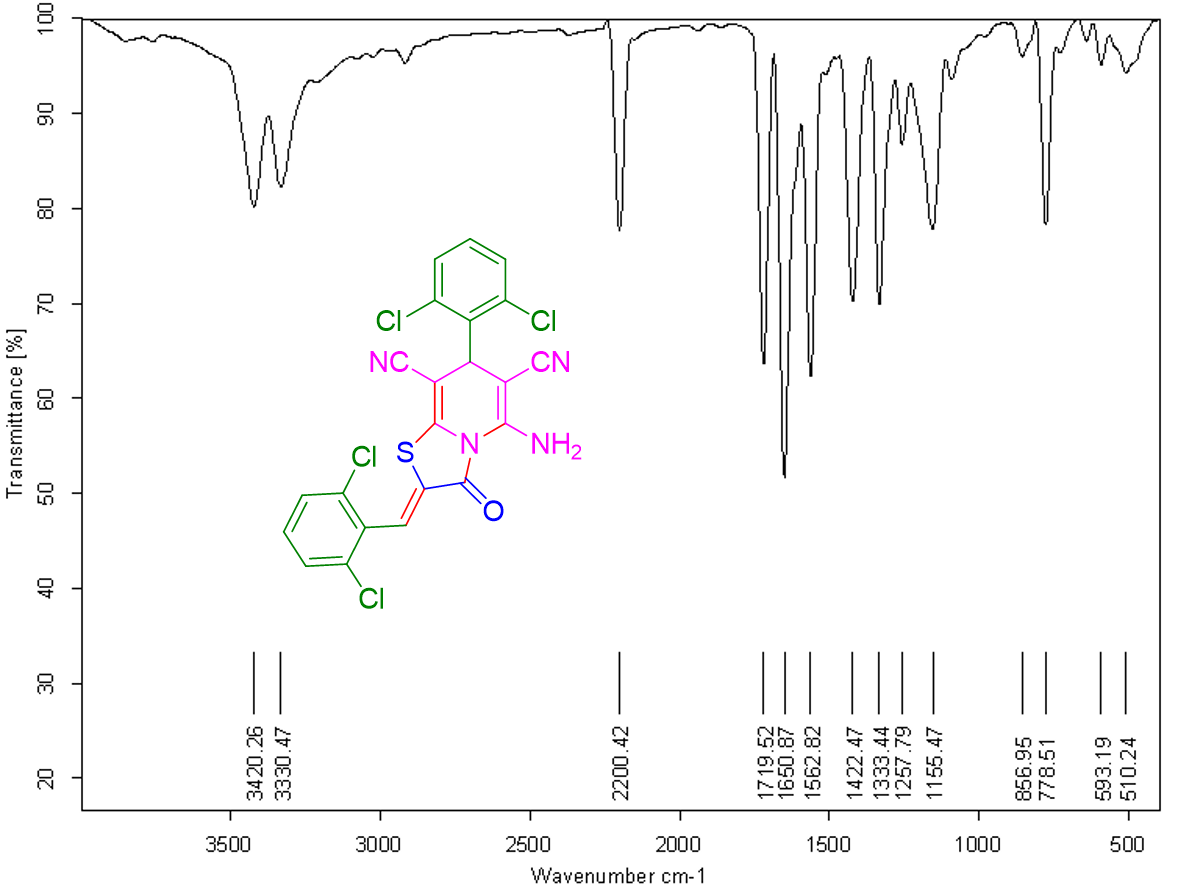


Figure 7. IR spectra of compound **4c**


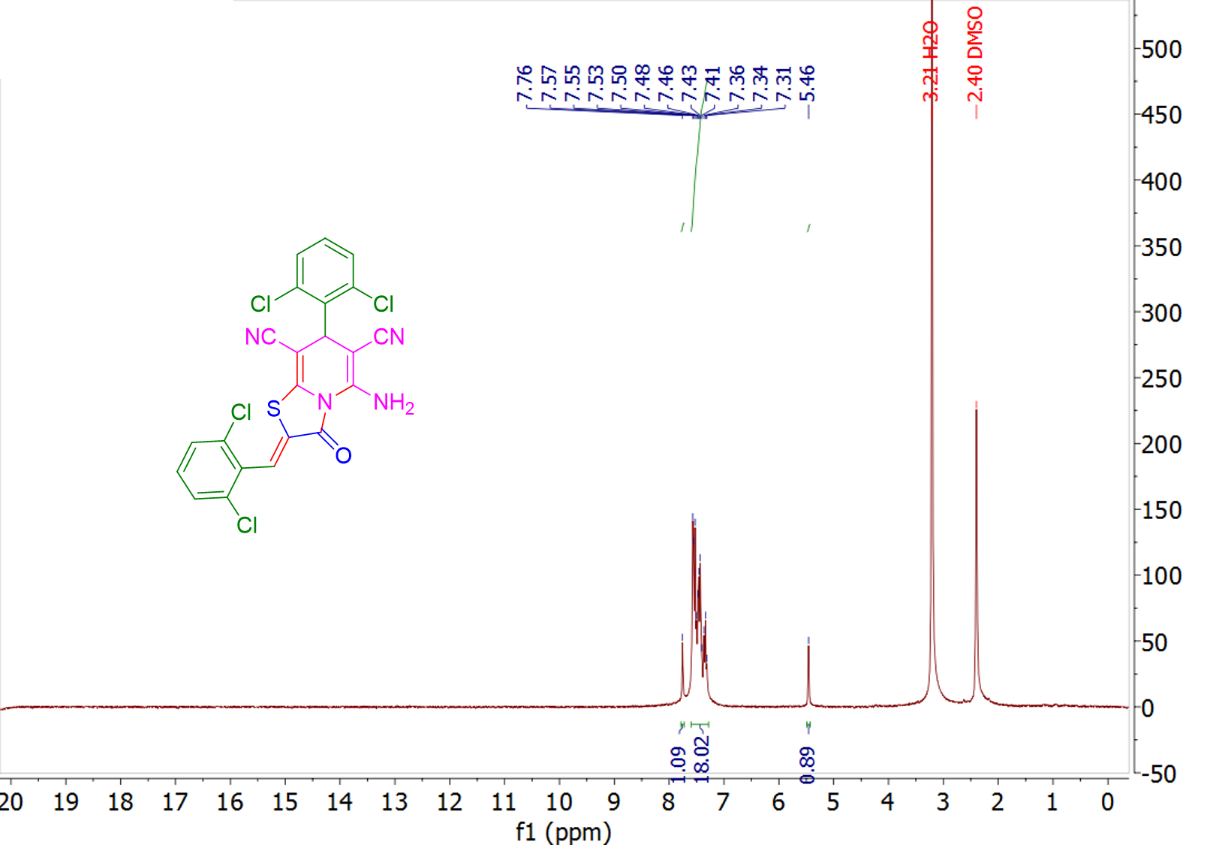


Figure 8. ^1^HNMR spectra of compound **4c**


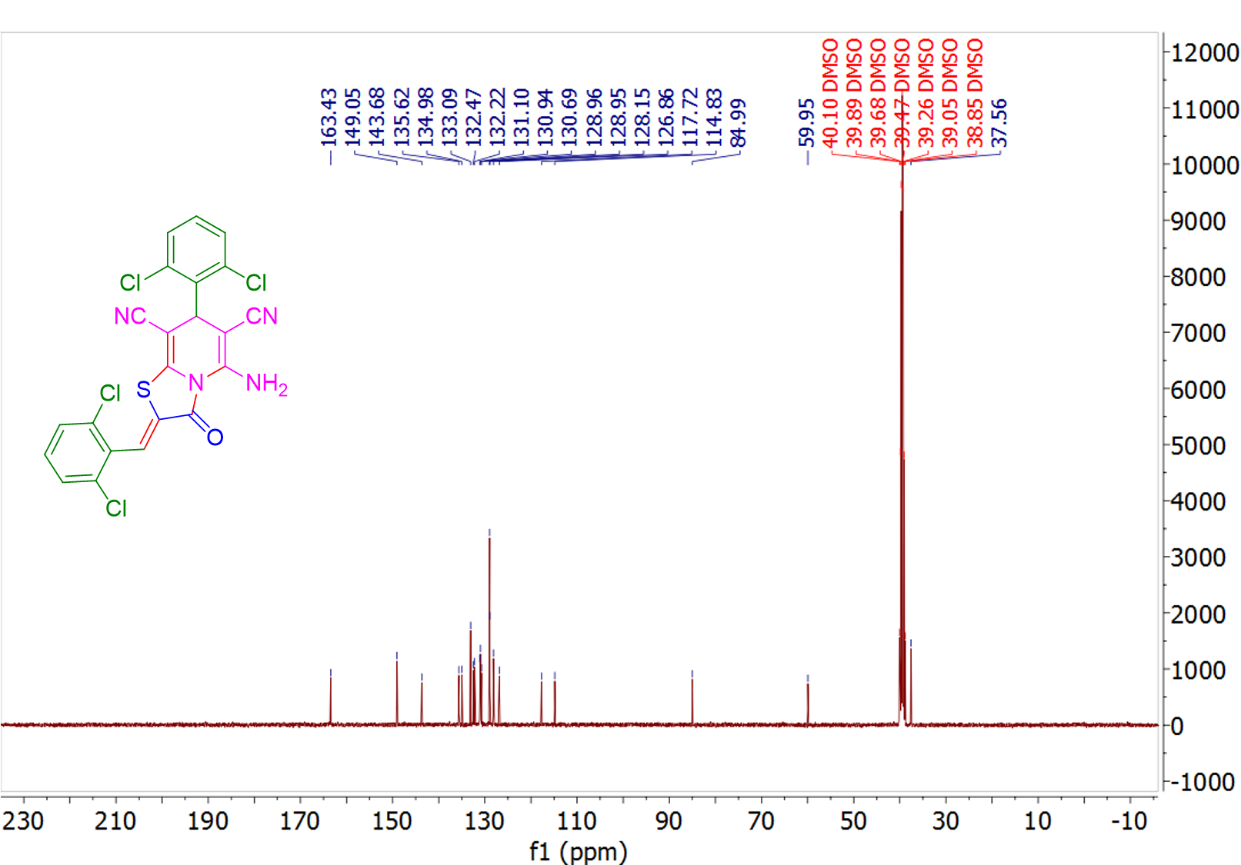


Figure 9. ^13^CNMR spectra of compound **4c**


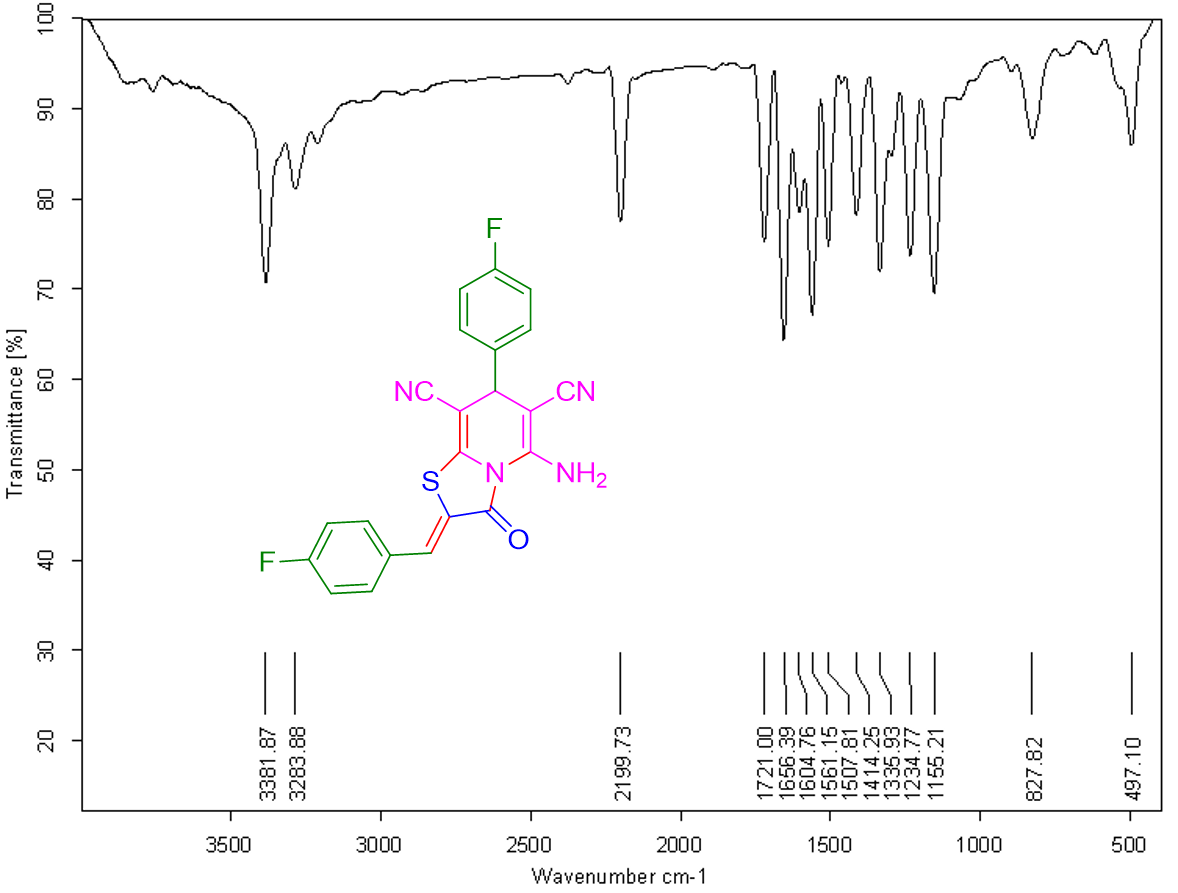


Figure 10. IR spectra of compound **4d**


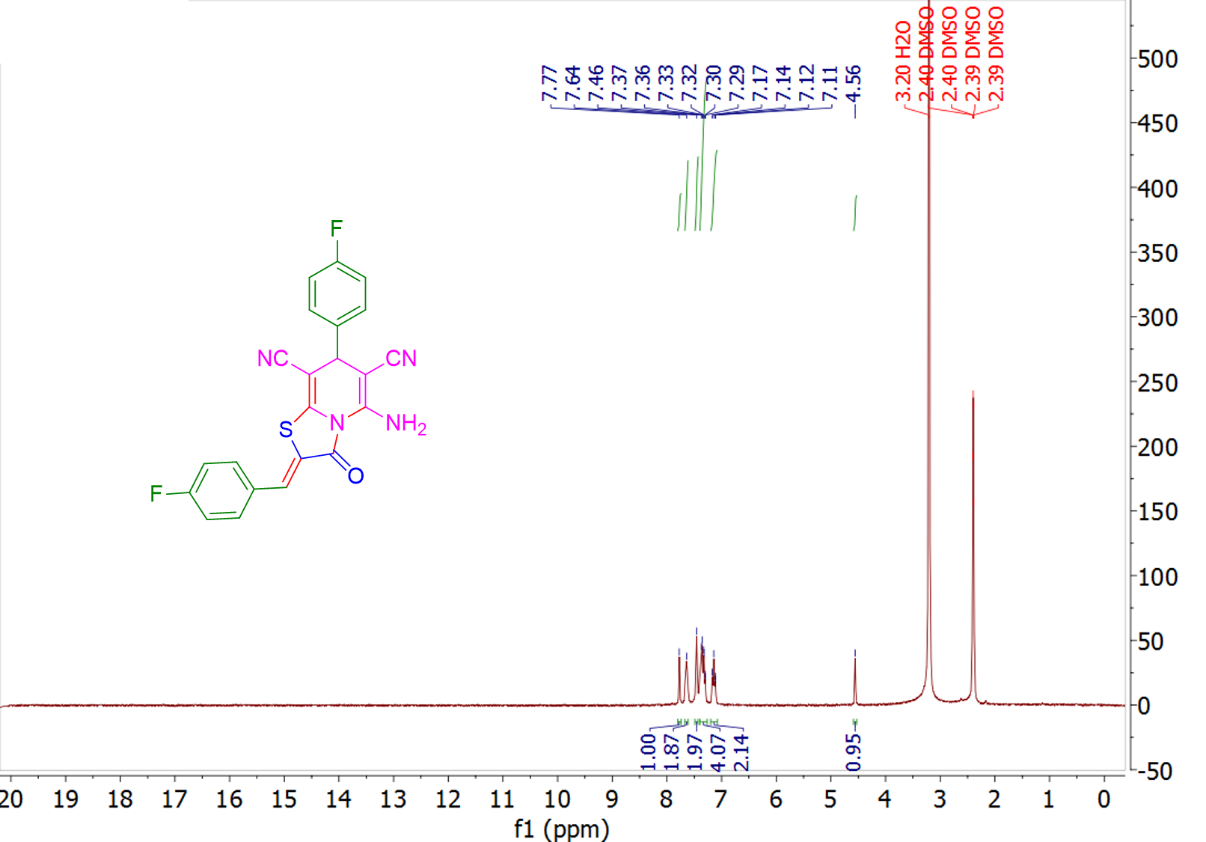


Figure 11. ^1^HNMR spectra of compound **4d**


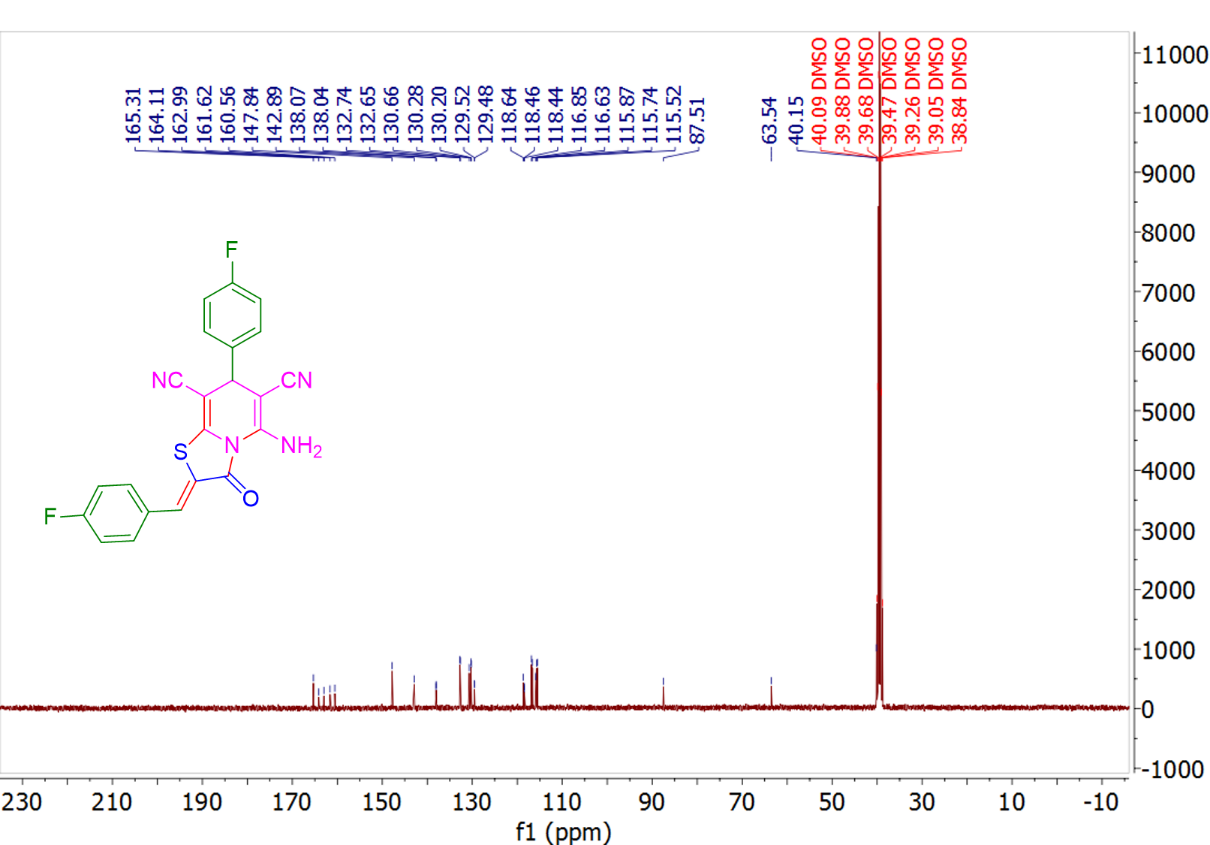


Figure 12. ^13^CNMR spectra of compound **4d**


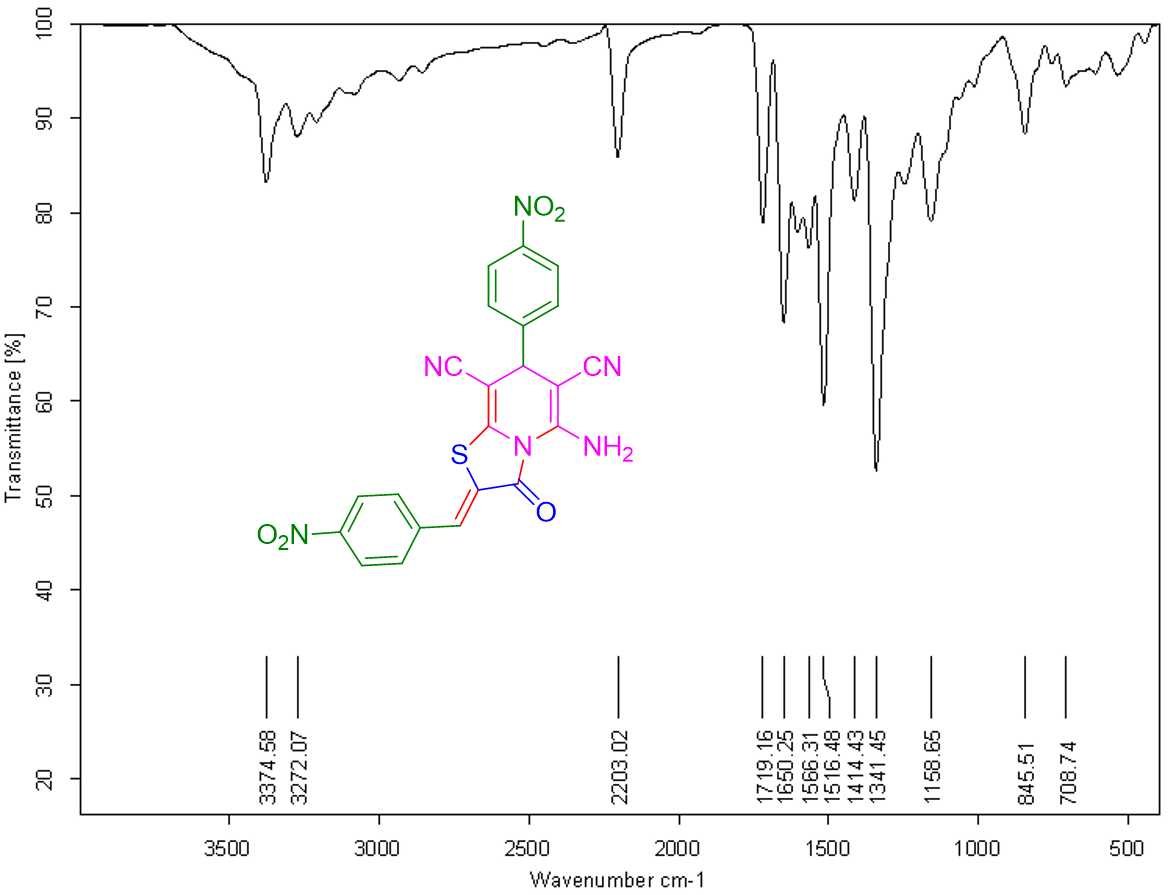


Figure 13. IR spectra of compound **4e**


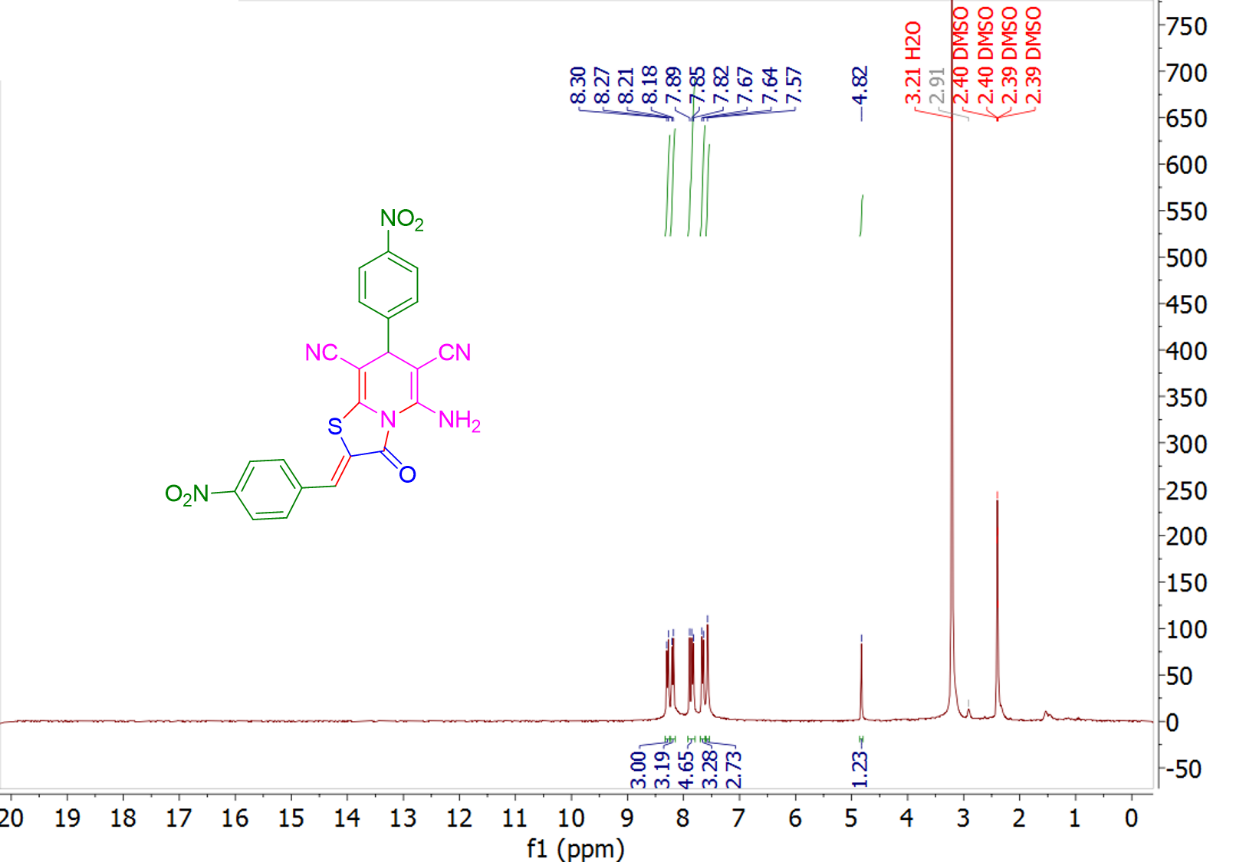


Figure 14. ^1^HNMR spectra of compound **4e**


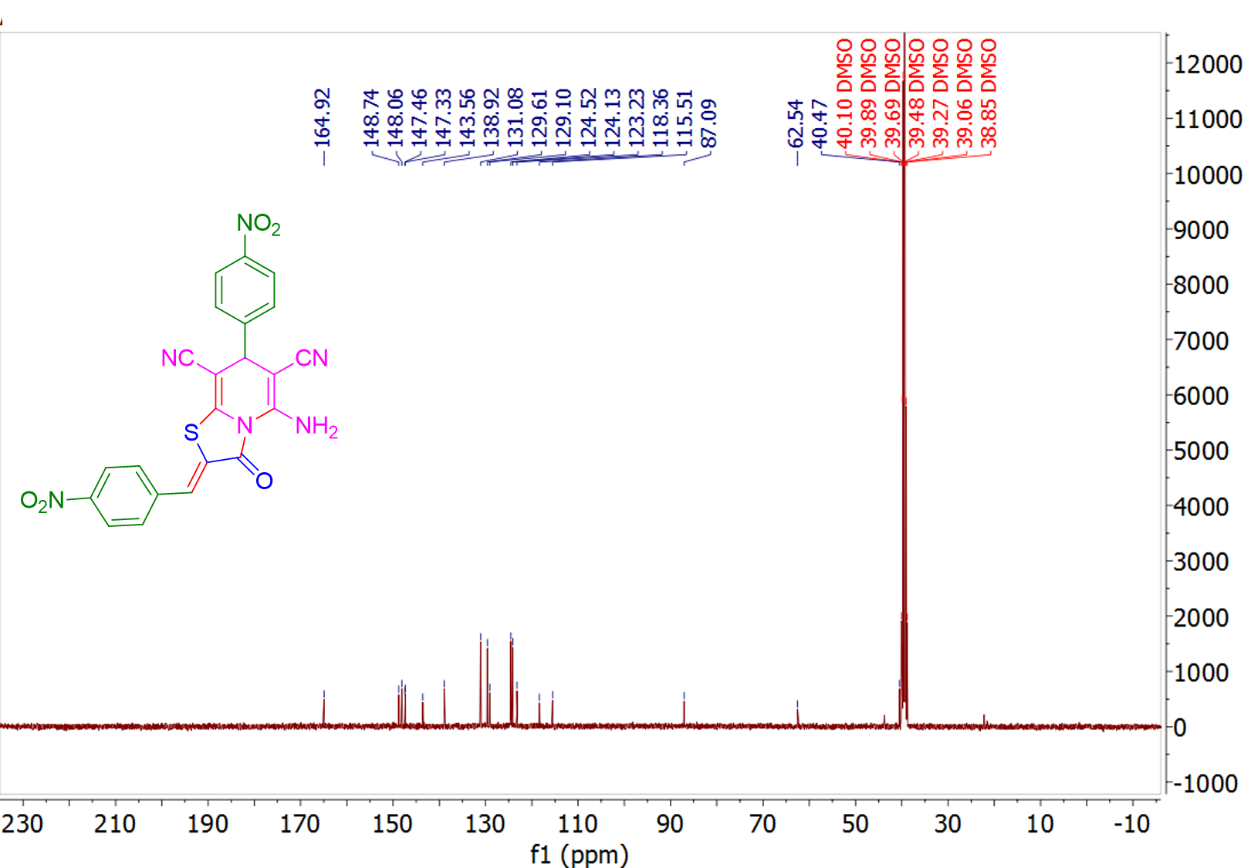


Figure 15. ^13^CNMR spectra of compound **4e**


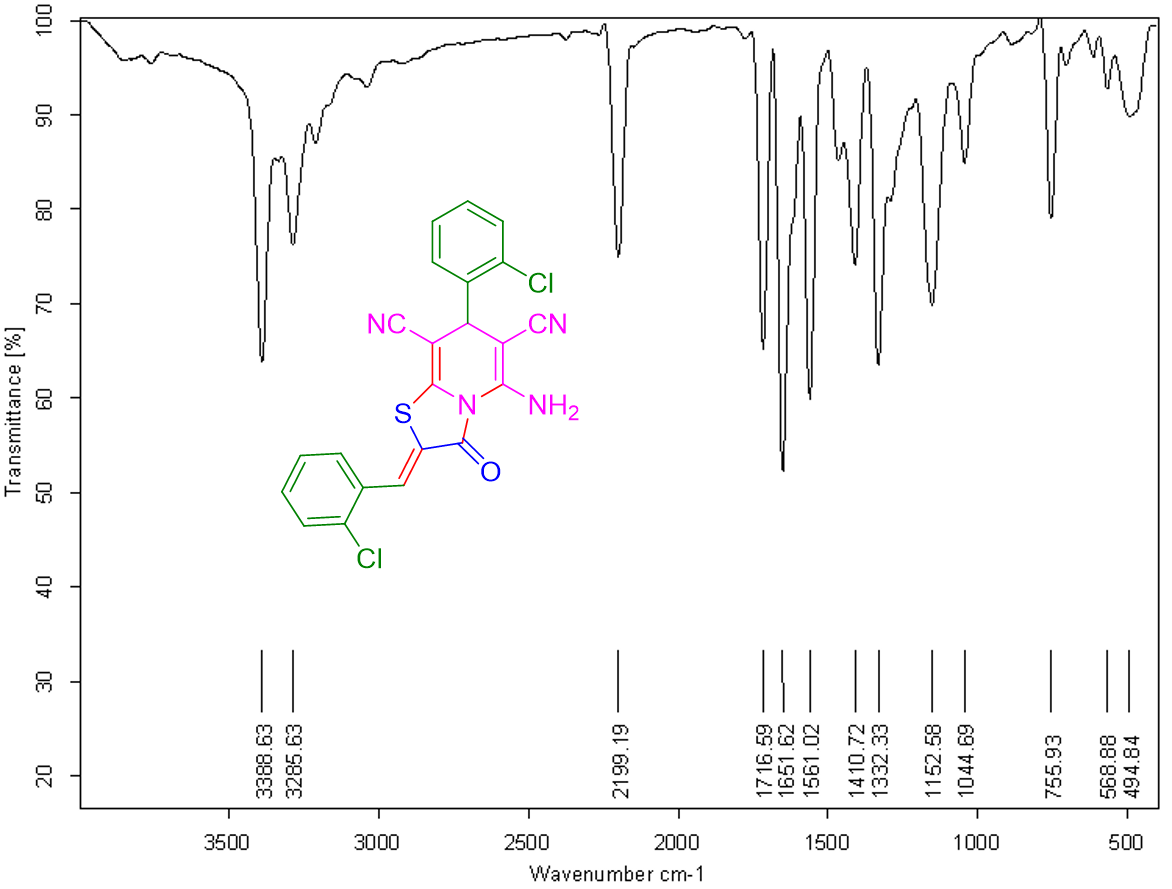


Figure 16. IR spectra of compound **4f**


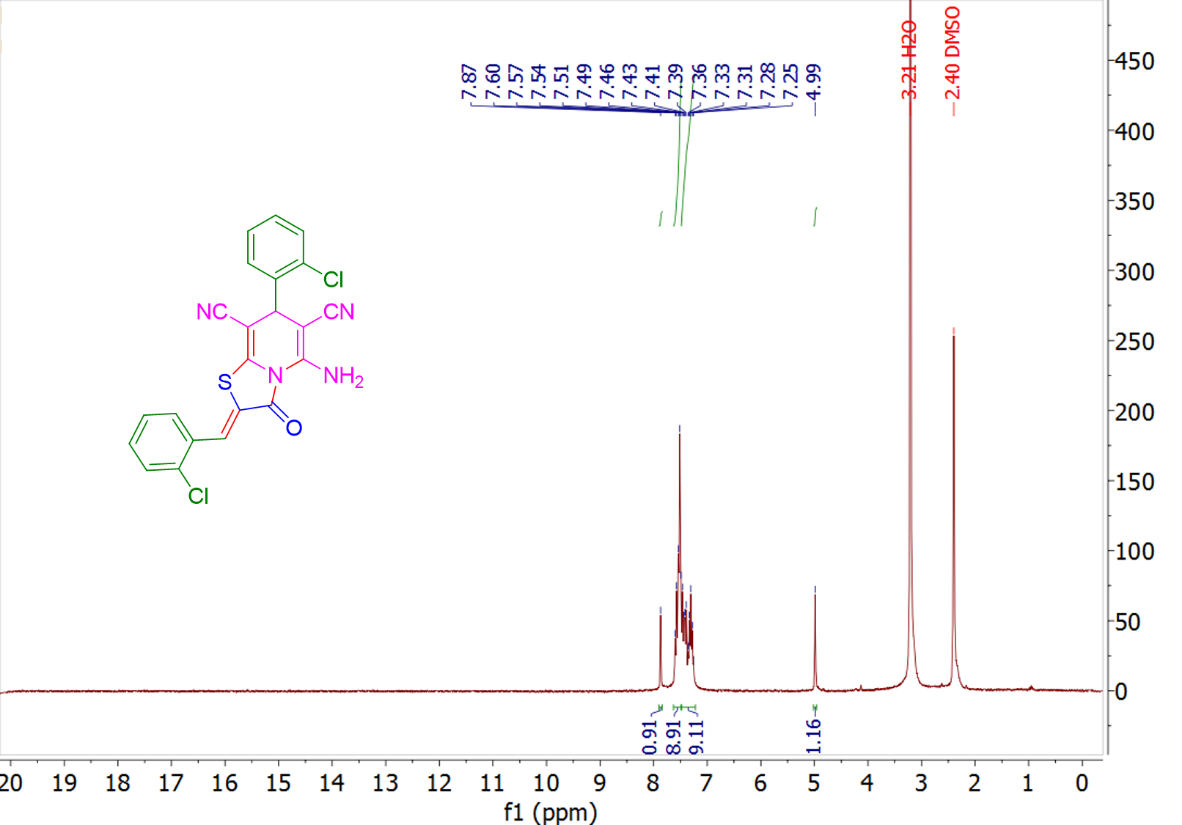


Figure 17. ^1^HNMR spectra of compound **4f**


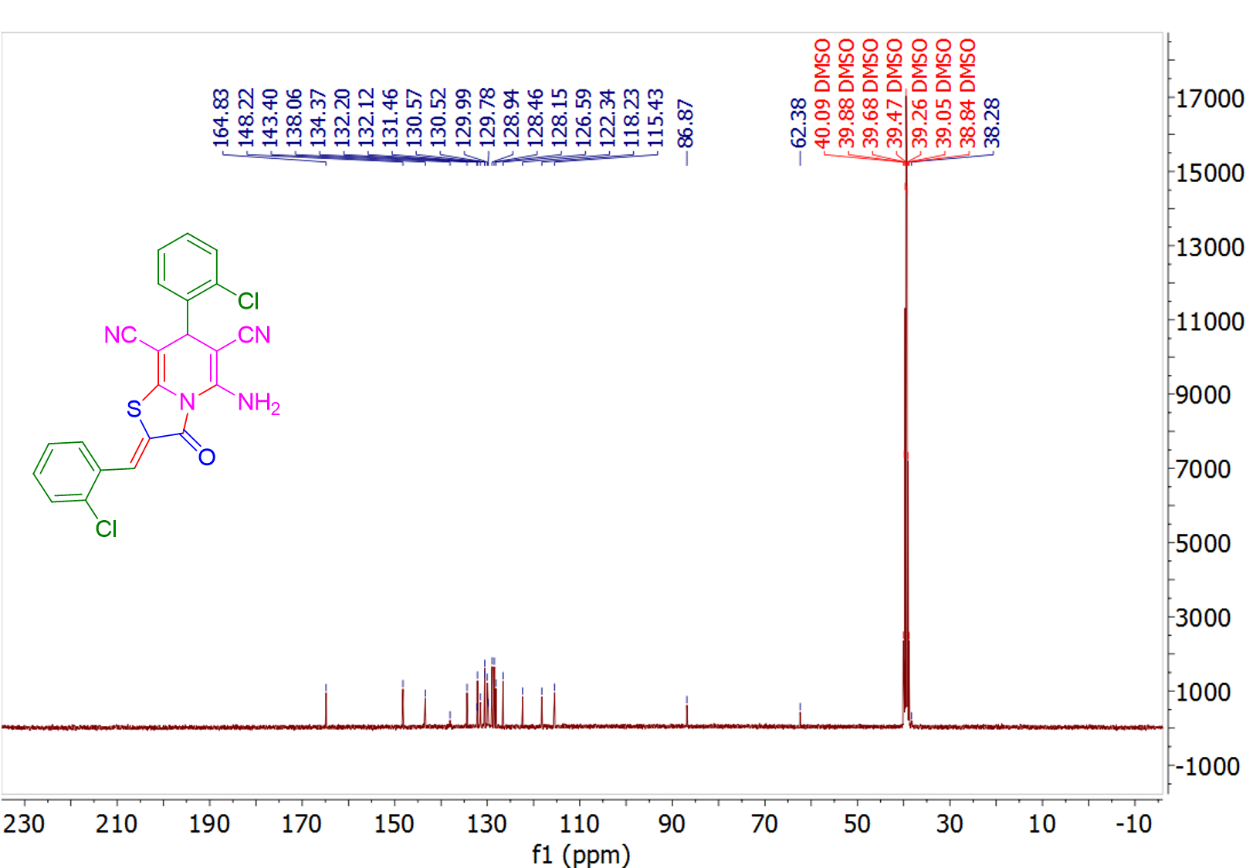


Figure 18. ^13^CNMR spectra of compound **4f**
